# The Association of Birth Weight and Infant Growth with Energy Balance-Related Behavior – A Systematic Review and Best-Evidence Synthesis of Human Studies

**DOI:** 10.1371/journal.pone.0168186

**Published:** 2017-01-12

**Authors:** Arend W. van Deutekom, Mai J. M. Chinapaw, Elise P. Jansma, Tanja G. M. Vrijkotte, Reinoud J. B. J. Gemke

**Affiliations:** 1 Department of Pediatrics, EMGO Institute for Health & Care Research, Institute for Cardiovascular Research VU, VU University Medical Center, Amsterdam, the Netherlands; 2 Department of Public and Occupational Health, EMGO institute for Health & Care Research, VU University Medical Center, Amsterdam, the Netherlands; 3 Department of Epidemiology and Biostatistics, EMGO institute for Health & Care Research, VU University Medical Centre, Amsterdam, Netherlands; 4 Department of Public Health, Academic Medical Centre, University of Amsterdam, Amsterdam, the Netherlands; McMaster University, CANADA

## Abstract

**Background:**

Suboptimal prenatal and early postnatal growths are associated with obesity in later life, but the underlying mechanisms are unknown. The aim of this study was to systematically review the literature that reports on the longitudinal association of (i) birth size or (ii) infant growth with later (i) energy intake, (ii) eating behaviors, (iii) physical activity or (iv) sedentary behavior in humans.

**Methods:**

A comprehensive search of MEDLINE, EMBASE, PsycINFO and The Cochrane Library was conducted to identify relevant publications. We appraised the methodological quality of the studies and synthesized the extracted data through a best-evidence synthesis.

**Results:**

Data from 41 publications were included. The quality of the studies was high in three papers, moderate in 11 and low in the large majority (n = 27) of papers appraised. Our best-evidence synthesis indicates that there is no evidence for an association of birth weight with later energy intake, eating behavior, physical activity or sedentary behavior. We found moderate evidence for an association of extreme birth weights (at both ends of the spectrum) with lower physical activity levels at a later age. Evidence for the association of infant growth with energy balance-related behavior was generally insufficient.

**Conclusions:**

We conclude that current evidence does not support an association of early-life growth with energy balance-related behaviors in later life, except for an association of extreme birth weights with later physical activity.

## Introduction

There is now an abundance of literature highlighting the importance of early life growth on adult-onset disease risk. Low birth weight, as a marker of suboptimal prenatal growth, is strongly linked to central obesity[1], cardiovascular disease[2] and type 2 diabetes.[3] In addition to low birth weight, high birth weight and accelerated postnatal weight gain have also been independently associated with cardiometabolic disease and obesity.[4] These associations may reflect physiological predictive adaptive responses to early life environmental cues, with long-term structural and/or functional changes that influence later health and disease risk.[5]

One of the hypothesized mechanisms underlying the association of pre- and early postnatal growth with later obesity and cardiovascular disease is the alteration of energy balance-related behaviors, including eating behavior, physical activity (PA) and sedentary behavior (SB). Several studies in rodents have shown that impaired prenatal nutrition followed by increased postnatal growth causes excessive intake and diminished PA in the offspring, preceding the development of obesity.[6–8] The hypothesis that early growth affects long-term energy balance regulation seems plausible as the hypothalamic neuro-endocrine circuits involved in energy homeostasis are highly sensitive for nutritional influences during gestation and directly after birth.[9] However, caution is needed when extrapolating findings from these rat studies to the human situation, because the timing in the development of energy-balance regulation is different between species and because the nutritional regimens used to model early life malnutrition are rather extreme, with intakes reduced to 30% of controls.[8] This might induce a pathological response not directly relevant to the normal human pregnancy.

Human data on the association of pre- and early postnatal growth with energy balance-related behaviors are limited and show conflicting results. For example, some studies found a positive association of birth weight with PA[10], whereas others reported no associations[11], inverse associations[12], PA-specific associations[13], age-specific associations[14] or gender-specific associations.[15] Factors that may contribute to the equivocal association of perinatal growth with energy balance-related behavior include differences in study population, the severity of growth retardation in low birth weight subjects, methodology pertaining to data collection (such as use of questionnaires) and different types of PA studied.

Reviews have summarized the association of pre- and early postnatal growth with either energy intake[16–18], or the combination of energy intake with PA[19–21]. The majority of reviews concluded that suboptimal pre- and early postnatal growth has negative effects on these behaviors in later life. However, these conclusions are mostly based on a non-systematic search of the literature, with evidence derived from animal models and the studies’ quality not taken into account. Øglund *et al*. reviewed the literature on the relation of perinatal growth with accelerometer-assessed PA in children and concluded that there is no evidence for an association of birth weight with childhood PA based on a formal meta-analysis.[22] However, the authors could only include five of nine identified studies in their meta-analysis because of the heterogeneity of the reported data. In addition, the methodological quality of the studies was not accounted for. A best-evidence synthesis overcomes these limitations by synthesizing all the available evidence and weighing the methodological quality of the studies.

In the present systematic review we assess the association of birth weight and infant growth with energy balance-related behaviors in children, adolescents and adults, including a quality assessment and data synthesis of evidence from human studies on the association of (i) birth size and/or (ii) infant growth with later (i) energy intake, (ii) eating behavior, (iii) PA levels and/or (iv) SB.

## Methods

### Literature search

A comprehensive systematic search was performed in the bibliographic databases PubMed, EMBASE.com, PsycINFO (via EBSCO) and The Cochrane Library (via Wiley) from inception to January 5^th^, 2016. Search terms included controlled terms from MeSH in PubMed and EMtree in EMBASE.com, thesaurus terms in PsycINFO as well as free text terms. In The Cochrane library only free text terms were applied. Search terms expressing perinatal growth (e.g., birth weight, infant growth, etc.) were used in AND-combination with terms representing ‘Energy balance-related behaviors’ (e.g., sedentary, intake, activity, etc.). If possible, the search was restricted by excluding non-relevant publication types (e.g., editorials, practice guidelines, biographies, etc.). The full search strategy is available as online supplement on the journal’s website (S2 File). Additionally, the reference lists of all selected articles and published reviews on this topic were screened for potentially relevant publications (backward citation tracking), and we used Science Citation Index to identify all the subsequent articles that cite any of the selected articles or relevant reviews (forward citation tracking).

### Eligibility criteria

Studies were included if they met the following criteria: (i) the study was a (historical or birth) cohort study; (ii) the study described at least one anthropometric measurement during birth or change in anthropometric measurements in infancy; and (iii) the dependent variable was a measure of energy intake, eating behavior, PA or SB. We excluded: (i) animal studies; (ii) studies reporting on the effects of an (nutritional) intervention or famine exposure; (iii) publications written in another language than English, German, French or Dutch; (iv) certain publication types: editorials, legal cases, interviews, etc.

### Definition of the outcome variables

The following definitions were used to help guide the eligibility assessment: energy intake is the amount of energy consumed as food (expressed in calories or joules) per unit of time (mostly day); eating behavior is the patterns of behaviors (thoughts, actions and intents) that a person enacts in order to regulate its energy intake[23]; PA is any bodily movement produced by skeletal muscles that requires energy expenditure, such as active transportation or participation in sports[24]; and SB refers to any waking activity characterized by an energy expenditure ≤ 1.5 metabolic equivalents and a sitting or reclining posture, such as sitting, watching TV, playing video games.[25] Eligible outcome measures included those obtained by objective measures (e.g., weighing of foods, observation of behaviors, or activity measurements by accelerometer) and self-/parent-reports (e.g., questionnaires asking about food intake, sport participation or screen time). The operational definition of each outcome (e.g., accelerometer cut points defining PA or SB) was acquired from each publication.

### Selection process

Two reviewers (AvD and EJ) independently screened all titles and abstracts of articles identified through the search process for eligibility. If necessary, the full text article was checked for the in- and exclusion criteria. Differences in judgment were resolved through discussion until consensus was reached. Full text of all eligible articles was obtained for further review.

### Data extraction

The following data were extracted using a structured form developed for this review (available upon request): (i) general characteristics of the article (author’s name, publication year), (ii) study characteristics (design, country), (iii) study population (number, percentage male, mean age at outcome), (iv) method of measurement (objective or self-report) and type of behavior studied, (v) relevant results including measures of associations where possible and (vi) confounders results were adjusted for.

### Quality assessment

Two authors (AvD and MC) independently assessed the methodological quality of all included studies, using a 10-item criteria list, adapted from the Effective Public Health Practice Project Quality Assessment Tool (see Table 1).[26] Of the original tool, we deleted three domains that were regarded irrelevant for the included studies. All the studies were cohort studies, so we deleted the domain ‘study design’. Further, ‘blinding’ and ‘intervention integrity’ were irrelevant for the observational studies, and therefore deleted from the tool. What remains are the five domains that we consider fundamental for an appropriate appraisal of the methodological quality: (i) selection bias, (ii) potential confounding, (iii) method of measurement, (iv) study attrition, and (v) data analysis. Each dimension was judged as strong, moderate or weak, based on predefined criteria. If the study referred to another publication describing the design, study population, psychometric properties of the measurements or other relevant information for the quality assessment, we retrieved the respective publication to score the dimension of concern. We defined high-quality studies as having at least two strong and no weak dimensions, moderate-quality studies as having less than two strong dimensions, but no more than one weak dimension, and low-quality studies as having more than one weak dimension.

**Table 1:** Criteria list for the quality assessment.

Dimension	Criteria	Judgment rules	
Selection bias	(Q1) Are the individuals selected likely to be representative of the target population?	Strong:	Q1 = 1 and Q2 = 1
	(1) very likely (e.g., randomly selected from target population), (2) somewhat likely (e.g., selected from a source); (3) not likely (e.g., self-referred); (4) can’t tell	Moderate:	(Q1 = 1 or 2) and (Q2 = 2 or 4)
	(Q2) What percentage of selected individuals agreed to participate?	Weak:	(Q1 = 3) or (Q2 = 3) or (Q1 = 4 and Q2 = 4)
	(1) 80–100% agreement; (2) 60–79% agreement; (3) less than 60% agreement; (4) not applicable, (5) can’t tell		
Confounding	(Q1) Were there important differences between groups?	Strong:	Q1 = 2 or Q2 = 1
	: (1) yes; (2) no; (3) can’t tell	Moderate	(Q1 = 1 or 3) and Q2 = 2
	(Q2) If yes, what were the relevant confounders that were controlled for?	Weak:	(Q1 = 1 or 3) and (Q2 = 3 or 4)
	(1) at least gestational age, sex and age; (2) at least gestational age; (3) not gestational age; (4) can’t tell		
Measurement	(Q1) Were tools to collect outcome data shown to be valid?	Strong:	Q1 = 1 and Q2 = 1
	(1) yes; (2) no; (3) can’t tell	Moderate:	(Q1 = 1) and (Q2 = 2 or 3)
	(Q2) Were tools to collect outcome data shown to be reliable?	Weak:	(Q1 = 2) or (Q1 = 3 and Q2 = 3)
	(1) yes (objective measures or questionnaires with ICC > 0.7 or Pearson > 0.8); (2) no; (3) can’t tell		
Study attrition	(Q1) Were withdrawals and drop-outs reported in terms of numbers and/or reasons per group?	Strong:	Q2 = 1
	(1) yes; (2) no; (3) not applicable (i.e. one time surveys or interviews); (4) can’t tell	Moderate:	Q2 = 2 or 4
	(Q2) Indicate the percentage of participants completing the study.	Weak:	(Q1 = 4) or (Q2 = 3 or 5)
	(1) 80–100%; (2) 60–79%; (3) less than 60%; (4) not applicable (i.e. retrospective); (5) can’t tell		
Data analysis	(Q1) The number of cases was at least 10 times the number of the independent variables.	Strong:	Q1 = 1 and Q2 = 1
	(1) yes; (2) no; (3) can’t tell	Moderate:	(Q2 = 2 or 3) or (Q3 = 2 or 3)
	(Q2) Point estimates and measures of variability are presented.	Weak:	(Q2 = 2 or 3) and (Q3 = 2 or 3)
	(1) yes; (2) no; (3) not applicable		

Criteria list, and the corresponding judgment rules for each dimension, for the assessment of the methodological quality of the studies included in this review adapted from the Effective Public Health Practice Project Quality Assessment Tool.[26]

### Best-evidence synthesis

The included studies were very heterogeneous, especially with regard to the type and measurement of behavior, the categorization of subjects and type of statistical analysis. Therefore, statistical pooling by means of a formal meta-analysis was not feasible, and we performed a best-evidence synthesis. A best-evidence synthesis is a systematic qualitative summarization of available evidence, which helps to reduce the chance of conflicting results and conclusions.[27] We stratified the best-evidence synthesis for studies that are similar with respect to the determinant (birth weight or infant growth) and type of behavior studied. For the best-evidence synthesis, we took the methodological quality into account according to the following decision rules: Strong evidence, provided by generally consistent results in at least two high-quality studies. Moderate evidence, provided by generally consistent results in one high-quality study and at least one moderate- or low-quality study, or generally consistent results in multiple moderate- or low-quality studies. Insufficient evidence, when less than two studies were available or inconsistent findings in multiple studies. Consistent evidence is defined as at least 75% of the findings with similar direction of effect. If there were at least two studies of high methodological quality, we disregarded the studies of low quality in the evidence synthesis; those studies were thus not incorporated in the conclusion.

## Results

### Literature search

**Fig 1:**
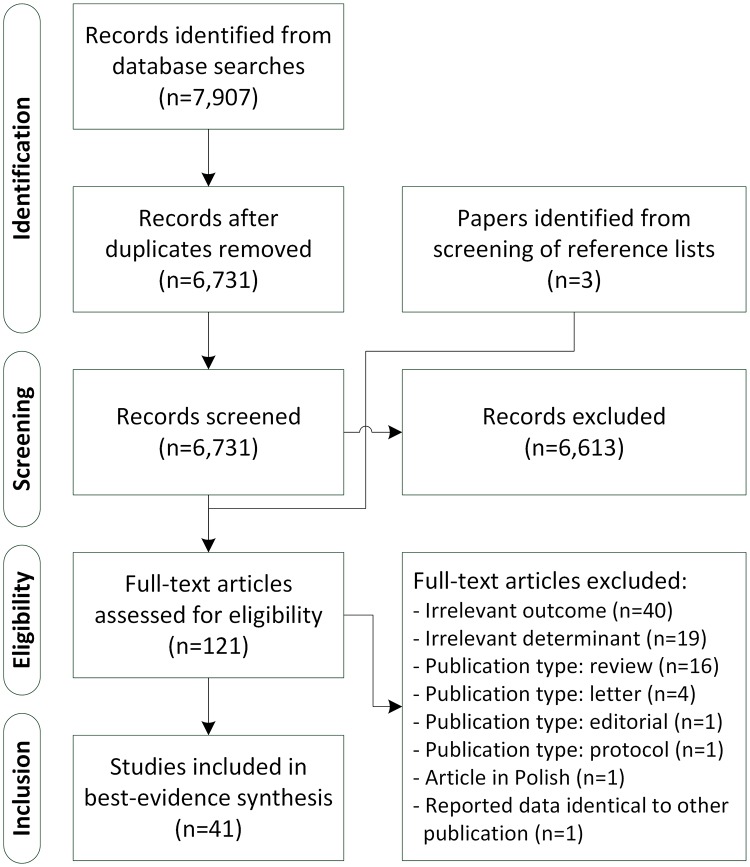
Flowchart of the search process and study selection.

The literature search generated a total of 7,907 references: 3,724 in PubMed, 3,061 in EMBASE.com, 577 in PsycINFO and 545 in The Cochrane Library. We consecutively removed duplicates that were selected from more than one database, excluded non-relevant articles by screening the titles and abstracts, and reviewed the remaining articles in full text. (see Fig 1) Eventually, 39 articles met the inclusion criteria and were eligible for further analysis.[10–15, 28–60] Three additional eligible papers[61–63] were identified through backward and forward citation tracking. Two papers reported exactly the same association based on the same data[56, 63], so the publication with the least additional details[63] was omitted for further analysis. Two studies[11, 47] had overlapping pooled data and partly focused on the same association (i.e., of birth weight with SB), so only the unique results (i.e., of birth weight with PA) of the least comprehensive study[11] was included in the analysis. Table 2 provides an overview of the characteristics of the 41 included studies.

**Table 2:** Summary of the studies reporting the association of pre- and postnatal growth with energy balance-related behavior in humans.

**Determinant**	**Author, publication year**	**Type of study**	**Population descriptives (n, % male, country)**	**Mean age at time of outcome assessment**	**Outcome (type and method of measurement)**	**Relevant result**	**Adjustment for confounders**
Normal birth weight	**Atladottir, 2000**[28]	Prospective cohort	N = 138, 51% male, Iceland	followed through first year of life	Energy intake (dietary record and weighing)	Birth weight was significantly associated with energy intake at the age of 2 months (r = 0.20, P<0.05), but not with energy intake at 4, 6, 9 or 12 months.	Sex.
	**Barbieri, 2009**[29]	Prospective cohort	N = 2,050, 48% male, Brazil	24y	Macronutrient intake (questionnaire)	BWR was not associated with total daily energy intake (P = 0.43).	GA, sex, BMI, smoking, education, PA, maternal education, maternal income, maternal smoking.
	**Boone-Heinonen, 2015**[30]	Prospective cohort	N = 3,353, 52% male, USA	13.5y	Macronutrient intake (questionnaire)	LBW (BW<2.5 kg) boys had a lower reported energy intake than NBW boys (mean [SE] kcal*day^-1^: 1981 [118] vs. 2360 [45], P<0.05). HBW (BW>4 kg) boys had a similar energy intake than NBW boys (2506 [129]). There was no significant difference in energy intake between LBW, NBW and HBW girls (1845 [88], 1904 [28], 1823 [92], respectively).	No.
	**Dubignon, 1969**[31]	Prospective cohort	N = 210, 48% male, Canada	Neonatal period	Intake of formula milk over first 4 days of life (direct observation)	Birth weight (ranked and grouped in quartiles) was positively associated with mean daily intake of formula milk in the first 4 days of life (mean ounces per day for increasing birth weight quartiles: 9.9 to 11.5, P_trend_<0.05).	No.
	**Li, 2015**[32]	Prospective cohort	N = 52,114, 0% male, USA	35.6y	Macronutrient intake (questionnaire)	Total energy intake was not different between ascending birth weight quintiles (mean [SE]: 1,791 [565], 1,779 [746], 1,800 [539], 1,806 [543] and 1796 [558] kcal*day^-1^, respectively).	No.
	**Perälä, 2012**[33]	Retrospective cohort	N = 1,797, 47% male, Finland	61.5y	Macronutrient intake (questionnaire)	Birth weight was not associated with energy intake (β = 221.0 kJ*day^-1^, 95%-CI: -140.6; 582.6).	GA, sex, age, BMI, education, smoking.
	**Ruiz-Narváez, 2014**[34]	Prospective cohort	N = 21,624, 0% male, USA	38.4y	Energy intake (questionnaire)	Birth weight (categorized as very low, <1,500g, low 1,500–2,499g, normal 2,500–3,999g, and high ≥4,000g) was positively associated with energy intake (mean intake for increasing birth weight categories: 1,493 to 1,516 kcal*day^-1^; P_trend_<0.001).	No.
	**Shultis, 2005**[35]	Prospective cohort	N = 1,278, 54% male, United Kingdom	8, 18 and 43 months and 7 years	Diet at age 8, 18, 43 months, and 7 years (dietary record)	There was no association of birth weight with mean daily energy intake at ages 8, 18, 43 months, or 7 years (β up to 18.07 kcal*day^-1^, 95%-CI: -3.72; 39.86).	GA, sex, age, SES, parental education, birth order, mid-parental height.
Extreme birth weight	**Kaseva, 2013**[61]	Prospective cohort*	VLBW adults, N = 151 vs. N = 156 controls, 39% male, Finland	22.5y	Mean daily energy intake (dietary record)	1. There was no difference in total daily energy intake between VLBW adults and NBW controls (P = 0.2)	Sex, age, BMI, height, SES, living at parents, smoking and maternal smoking during pregnancy.
	**Ounsted, 1975**[37]	Prospective cohort	N = 191, 54% male, United Kingdom	2 mo	Milk intake (direct observation)	SGA infants consumed more milk per kg body weight, than AGA infants (mean [SD]: 192.4 [37.5] vs. 161.2 [37.2] cc*kg^-1^). LGA infants consumed less than AGA infants (142.0 [27.1] cc*kg^-1^, all P<0.05).	No.
Other birth size	**Perälä, 2012**[33]	Retrospective cohort	N = 1,797, 47% male, Finland	61.5y	Macronutrient intake (questionnaire)	PI was not associated with energy intake (β = 22.0 kJ*day^-1^, 95%-CI: -18.2; 92.2).	GA, sex, age, BMI, education, smoking.
Infant growth	**Atladottir, 2000**[28]	Prospective cohort	N = 138, 51% male, Iceland	followed through first year of life	Energy intake (dietary record and weighing)	Relative growth from birth to 12 months was positively associated with energy intake per kg body weight at the age of 12 months (r = 0.30, P<0.01)	Sex.
Short statue after SGA	**Boonstra, 2006**[38]	Retrospective part of intervention study	Children with short statue after SGA. N = 88, 41% male, the Netherlands	5.9y	Macronutrient intake (questionnaire)	Children with short stature born SGA had a significantly lower mean (SD) energy intake compared to the recommended-daily intake of age-matched children (1,337 [309] vs. 1,697 [237] kcal, P<0.001).	No.
Eating behavior							
Determinant	**Author, publication year**	**Type of study**	**Population descriptives (n, % male, country)**	**Mean age at time of outcome assessment**	**Outcome (type and method of measurement)**	**Relevant result**	**Adjustment for confounders**
Normal birth weight	**Brown, 2012**[39]	Retrospective observational study	N = 298, % male unknown, United Kingdom	18–24 months	Satiety responsiveness and food responsiveness (questionnaire)	No significant association was seen of birth weight with satiety responsiveness or food responsiveness at 18–24 months (data not shown).	Weight, maternal age, maternal education, maternal BMI.
	**Cardona Cano, 2015**[40]	Prospective cohort	N = 3,227, 50% male, the Netherlands.	1.5y, 3y and 6y	Picky eating (questionnaire)	Birth weight was inversely associated with the odds of being a persistent picky eater, with a relative risk ratio of 0.54 per kg increase in birth weight (95%-CI: 0.35; 0.82).	Sex, ethnicity, birth order, maternal age, maternal BMI, maternal education, maternal income, maternal smoking during pregnancy.
	**Escobar, 2014**†[41]	Prospective cohort	N = 196, 52% male, Canada	4y	Emotional overeating (questionnaire)	Children with BWR < 0.85 had a similar emotional overeating score than the other children (difference: 0.40, 95%-CI: -0.64; 1.43).	GA, sex, BMI, mother-child interaction.
	**Migraine, 2013**[42]	2 prospective cohorts	N = 479, 53% male, France	2y	Drive-to-eat score (questionnaire)	Birth weight was inversely associated with drive-to-eat score in the preterm cohort (P = 0.001), but not in the term cohort (P = 0.10).	GA, sex, duration of breast feeding, maternal age, maternal BMI, maternal education.
	**Oliveira, 2015**[43]	3 prospective cohorts	N = 577–6279, % male not given, UK, Portugal and France	4–6mo, 12–15mo, 24mo and 48–56mo	Feeding difficulties, poor eating, food refusal, difficulties in establishing a daily routine (questionnaire)	70 potential associations were assessed between categories of birth weight (<p10 and >p90; p10-p90 reference) and eating behaviors (4 categories) by age at outcome (4 categories) and cohort (3 categories). There were four significant associations. In the Portuguese cohort, a birth weight under p10 was associated with feeding difficulties (OR 1.73, 95%-CI: 1.09; 2.75) and poor eating (OR 1.98, 95%-CI: 1.98; 2.88) at 4–6mo only and difficulties in establishing a daily routine at 48–54 mo only (OR 1.67, 95%-CI: 1.21; 2.2.31). In the British cohort a birth weight under p10 was associated with feeding difficulties at 4–6mo only (OR 1.26, 95%-CI: 1.05; 1.51).	GA, sex, BMI, type of birth, duration of breastfeeding, number of older siblings, maternal age, maternal BMI, maternal smoking during pregnancy, maternal education.
	**Silveira, 2012**†[44]	Prospective cohort	N = 160, 52% male, Canada	36mo	Impulsive eating (snack delay test)	In girls, but not in boys, a BWR < 0.85 was associated with a lower score on the snack delay test compared to other girls (mean [SE]: 7.76 [0.34] vs. 8.18 [0.13]), no P-value given.	GA, sex, IUGR-status, trial number.
Physical activity							
Determinant	**Author, publication year**	**Type of study**	**Population descriptives (n, % male, country)**	**Mean age at time of outcome assessment**	**Outcome (type and method of measurement)**	**Relevant result**	**Adjustment for confounders**
Normal birth weight	**Andersen, 2009**[45]	Meta-analysis of 13 cohorts	N = 43,482, 57% male, Nordic countries	Range 14–66y	Leisure time PA (questionnaire)	Compared with the reference category (3.26–3.75 kg), subjects in the birth weight categories 1.26–1.75, 1.76–2.25, 2.26–2.75, and 4.76–5.25 kg had a lower probability of undertaking leisure time PA, with odds ratios of 0.67 (95%-CI: 0.47; 0.94), 0.72 (0.59; 0.88), 0.89 (0.79; 0.99), and 0.65 (0.50; 0.86), respectively.	GA, sex, age, BMI, educational level, smoking.
	**Barbieri, 2009**[29]	Prospective cohort	N = 2,050, 48% male, Brazil	24y	PA level (questionnaire)	BWR was not associated with the prevalence of inactivity in women (P = 0.30) or in men (P = 0.18).	GA, sex, BMI, smoking, education, PA, maternal education, maternal income, maternal smoking.
	**Boone-Heinonen, 2015**[30]	Prospective cohort	N = 3,353, 52% male, USA	13.5y	PA level (questionnaire)	There was no significant difference in reported MET hours per week between LBW (BW<2.5 kg), NBW and HBW (BW>4 kg) adolescents (mean [SE]: 21.0 [1.7], 20.7 [1.1] and 19.0 [2.8] for LBW, NBW and HBW boys, respectively, and 29.9 [3.8], 28.0 [1.2], 28.1 [3.1] for girls).	No.
	**Campbell, 2010**[46]	Prospective cohort	N = 284, 44% male, Jamaica	13.4y	PA level (accelerometry)	Birth weight was not associated with mean c.p.m., (r = -0.081, P = 0.2) or percentage above 200 c.p.m. (r = -0.087, P = 0.1).	Sex, age, weight, height, pubertal stage.
	**Davies, 2006**[10]	Retrospective cohort	N = 24,874, 70.8% male, United Kingdom	38.0y	% of subjects undertaking regular PA (questionnaire)	Birth weight was positively associated with the likelihood of undertaking regular PA in adulthood (P = 0.02).	No.
	**Eriksson, 2004**[15]	Retrospective cohort	N = 500, 37% male, Finland	69.6y	Exercise frequency and intensity, yearly energy expenditure on exercise (questionnaire)	In men, but not in women, birth weight was inversely associated with exercise frequency (P = 0.009, effect size not given).	Age, BMI.
	**Gopinath, 2013**[14]	Prospective cohort	N = 1,794, 49% male, Australia. Resurvey at 17–18y: n = 1,213	12.7y	Time spent in MVPA (questionnaire)	Birth weight (ranked and grouped in quartiles) was positively associated with total MVPA (mean hours per week for increasing birth weight quartiles: 5.64 to 6.34; P_trend_ = 0.02) and outdoor MVPA (4.42 to 5.30; P_trend_ = 0.02) among 12-year-old children. At a resurvey at 17–18 years, birth weight was non-significantly positively associated with an increase in total MVPA and outdoor MVPA (P = 0.26 and P = 0.08, respectively).	GA, sex, age, ethnicity, BMI, parental education, home ownership, exposure to passive smoking.
	**Hallal, 2006**[62]	Prospective cohort	N = 4,453, 49% male, Brazil	10–12y	PA level, % of inactive subjects, defined as <300 min of PA per week (questionnaire)	The percentage of inactive subjects did not differ between subjects grouped in ascending birth weight tertiles (61.9%, 58.1% and 57.5%, respectively, P = 0.23). There was a borderline significant positive association between birth weight tertiles and amount of PA per week (210min, 234min and 240min, P = 0.05).	No.
	**Kehoe, 2012**[48]	Prospective cohort	N = 415, 49% male, India	7.5y	PA level (accelerometry)	Birth weight was not associated with mean c.p.m. (β = 9.62 c.p.m./kg, 95%-CI: -24.73; 43.96).	GA, sex, age, SES, body fat
	**Li, 2015**[32]	Prospective cohort	N = 52,114, 0% male, USA	35.6y	PA level (questionnaire)	Time in MVPA was not different between ascending birth weight quintiles (mean [SE]: 2.5 [3.8], 2.5 [3.9], 2.5 [3.9], 2.5 [2.8] and 2.8 [4.8] hours*week^-1^, respectively).	No.
	**Mattocks, 2008**[49]	Prospective cohort	N = 5,451, 48% male, United Kingdom	11.8y	PA level (accelerometry)	Birth weight was not associated with mean c.p.m. (β = -0.4 c.p.m.*100g^-1^, 95%-CI: -6.3; 5.5).	GA, sex, age, maternal education, SES.
	**Pahkala, 2010**[50]	Prospective cohort	N = 346, 59% male, Finland	13y	PA level (questionnaire)	Adolescents in the least active tertile had a birth weight similar to those in the most active tertile (mean [SE]): 3,487 [497] vs. 3,456 [437] for girls and 3,655 [555] vs. 3,637 [490] for boys).	Sex, weight, height, BMI, waist circumference, energy intake.
	**Pearce, 2012**[51]	Prospective cohort	N = 339, 50% male, United Kingdom	8–10y	PA level (accelerometry)	There was no significant association of standardized birth weight with total accelerometry count (r = -0.024, P>0.05) or MVPA (r = 0.016, P>0.05).	Sex, season of measurement.
	**Ridgway, 2011**[11]	Meta-analysis of four cohorts	N = 4,170, 44% male, Europe and Brazil	10.2–14.5y	PA level (accelerometry)	There was no significant association of birth weight with mean c.p.m. (β: -1.9 c.p.m.*kg^-1^, 95%-CI: -12.9; 9.2) or time in MVPA (0.6 min*day*kg^-1^, 95%-CI: -1.0; 2.1).	Sex, age, BMI, SES.
	**Ruiz-Narváez, 2014**[34]	Prospective cohort	N = 21,624, 0% male, USA	38.4y	Frequency of vigorous exercise (questionnaire)	Birth weight was not associated with frequency of vigorous exercise (P>0.05)	No.
	**Said-Mohamed, 2012**[52]	Retrospective cohort	N = 162, 56% male, Cameroon	4.1y	PA level (accelerometry)	Birth weight was not associated with total PA (β: -0.035 c.p.m.*kg^-1^, 95%-CI: -0.204; 0.134).	Sex, age, body composition.
	**Salbe, 1998**[53]	Retrospective cohort	N = 88 (of which 24 of diabetic mothers), 50% male, USA	5.5y	PA level (ratio of total energy expenditure [doubly labeled water method] to rest metabolic rate [ventilated hood]).	Although birth weight was higher in children of diabetic than of non-diabetic women (mean [SD]: 3.8 [0.6] vs. 3.5 [0.4] kg, P = 0.03), there was no difference in PA level (1.40 [0.12] vs. 1.38 [0.12]).	No.
	**Van Deutekom, 2015**[54]	Prospective cohort	N = 194, 54% male, the Netherlands	8.7y	PA level (accelerometry)	Birth weight was not related to time in MVPA (β = -1.93 min*day^-1^*SD^-1^; 95%-CI: -4.53; 0.67).	GA, Sex, age, SES, parental height and BMI, breast feeding, smoking during pregnancy.
	**Wijtzes, 2013**[55]	Prospective cohort	N = 347, 52.4% male, the Netherlands	2.1y	PA level (accelerometry)	Birth weight <2,500g was not associated with percentage of monitored time in MVPA (β = —1.2, 95%-CI: -2.6; 0.2) or mean c.p.m. (β = —77.7, 95%-CI: -177.6; 22.3), compared to birth weight >2,500g.	GA, sex, age, motor development, season of measurement, breast feeding, maternal BMI, number of siblings, daycare attendance, household income.
Extreme birth weight	**Hack, 2012**[12]	Prospective cohort	ELBW children, N = 168 vs. 115 controls, 36% male, USA	14.8y	PA level (questionnaire)	ELBW subjects had a significantly lower mean (SD) PA score, compared to NBW controls (2.56 [1.0] vs. 3.05 [0.91], P<0.001).	Sex, ethnicity, SES.
	**Kajantie, 2010**[56]	Prospective cohort*	VLBW adults, N = 136 vs. N = 188 controls, 41% male, Finland	22.3y	PA level, divided in occupational, commuting,leisure-time non-conditioning, and leisure-timeconditioning PA (questionnaire)	VLBW subjects reported less leisure-time conditioning PA than NBW controls (35.0% vs. 25.0% reporting “not much”, 38.0% vs. 25.0% reporting light activity, 22.1% vs. 41.5% reporting brisk activity, P_trend_ = 0.0002). VLBW adults report lower frequency (P_trend_ = 0.04) and intensity (P_trend_<0.0001) of PA and shorter average duration of PA sessions (P_trend_<0.0001). There was no difference in occupational, commuting, or leisure-time non-conditioning PA.	Sex, age, height, lean body mass, body fat percentage, smoking, SES, maternal smoking during pregnancy.
	**Kaseva, 2012**[13]	Prospective cohort*	VLBW adults, N = 94 vs. N = 101 controls, 41% male, Finland	25.0y	PA level, divided in occupational, commuting,leisure-time non-conditioning, and leisure-timeconditioning PA, energy expenditure (questionnaire)	VLBW subjects reported less leisure-time conditioning PA than NBW controls, including frequency (mean difference: -38.5%, 95%-CI: -59.8; -7.7), total time (-47.4, 95%-CI: -71.2; -4.1), total volume (-44.3%, 95%-CI -65.8; -9.2) and associated energy expenditure (-55.9%, 95%-CI: -78.6; -9.4). There was no difference in non-conditioning leisure-time PA, commuting PA, high intensity PA and total PA.	Sex, age, BMI, smoking, SES, personality traits.
	**Kaseva, 2015**[57]	Prospective cohort*	VLBW adults, N = 57 vs. N = 47 controls, 36% male, Finland	24.7y	PA level (accelerometry)	Between VLBW and NBW adults, there was no difference in daily PA (mean difference: -18.9 c.p.m., 95%-CI: -77.3; 39.5).	Sex, age, BMI, season of measurement, smoking, parental education.
	**Rogers, 2005**[58]	Prospective cohort	ELBW adolescents, N = 53 vs. 31 controls, 41% male, Canada	17.5y	Frequency of sport participation, frequency of PA (questionnaire)	ELBW subjects reported less sport participation than NBW controls (34% vs. 74%, P<0.001), and a lower frequency of PA (P<0.001)	Sex, ethnicity, SES.
	**Said-Mohamed, 2012**[52]	Retrospective cohort	N = 162, 56% male, Cameroon	4.1y	PA level (accelerometry)	Within the range of birth weight > 4.2 kg (n = 11), birth weight is negatively correlated with the time spent in MVPA (r:-0.8, p<0.001).	Sex, age, body composition.
Other birth size	**Eriksson, 2004**[15]	Retrospective cohort	N = 500, 37% male, Finland	69.6y	Exercise frequency and intensity, yearly energy expenditure on exercise (questionnaire)	In men, but not in women, PI was inversely associated with exercise frequency (P = 0.033), exercise intensity (P = 0.030) and energy expenditure on PA (P = 0.005, effect sizes not given).	Age, BMI.
	**Gopinath, 2013**[14]	Prospective cohort	N = 1,794, 49% male, Australia. Resurvey at 17–18y: n = 1,213	12.7y	Time spent in MVPA (questionnaire)	There were no significant associations of either birth length or head circumference with MVPA.	GA, sex, age, ethnicity, BMI, parental education, home ownership, exposure to passive smoking.
	**Kehoe, 2012**[48]	Prospective cohort	N = 415, 49% male, India	7.5y	PA level (accelerometry)	Neither birth length, nor head circumference was associated with mean c.p.m. (β = -4.48, 95%-CI: -11.41; 2.45, per cm birth length; β = -1.06, 95%-CI: -10.46; 12.58, per cm head circumference).	GA, sex, age, SES, body fat
	**Laaksonen, 2003**[59]	Retrospective cohort	N = 462, 100% male, Finland	50.6y	PA level (questionnaire)	PI was not associated with duration of strenuous leisure time PA (P_trend_ = 0.47) (data not shown).	No.
	**Mattocks, 2008**[49]	Prospective cohort	N = 5,451, 48% male, United Kingdom	11.8y	PA level (accelerometry)	Neither PI nor head circumference was associated with mean c.p.m. (β = 1.0, 95%-CI: -3.8; 5.9, per kg*m^-3^ PI; β = -3.5, 95%-CI: -9.2; 2.2, P = 0.2 per cm head circumference).	GA, sex, age, maternal education, SES.
Infant growth	**Hallal, 2006**[47]	Prospective cohort	N = 4,453, 49% male, Brazil	10–12y	PA level, % of inactive subjects, defined as <300 min of PA per week (questionnaire)	The percentage of inactive subjects did not differ between subjects grouped in ascending mean weight gain at 1–4 years quartiles (58.0%, 57.1%, 58.9% and 58.9%, P = 0.52) and mean weight gain at 4–11 years quartiles (61.2%, 54.7%, 55.8%, 61.4%, P = 0.58). There was a borderline significant inverse association between mean weight gain at 0–1 year quartiles and percentage of inactive subjects (61.0%, 61.3%, 58.5% and 53.7%, P = 0.09).	No.
	**Hallal, 2012**[60]	Prospective cohort	N = 457, 52% male, Brazil	13.3y	PA level (accelerometry)	Standardized weights at different ages from birth to age four were unrelated to total PA (counts per day). Standardized height at 3 and 12 months were inversely related to total PA (β = -18.0; 95%-CI: -33.0; -2.9, for 3 months. β = -23.4; 95%-CI: -39.7; -7.4, for 12 months).	GA, sex, family income, maternal education, maternal BMI, maternal smoking during pregnancy, all other weight and height variables.
	**Robinson, 2013**[36]	Retrospective cohort	N = 3,217, 52% male, United Kingdom	66.1y	PA score (questionnaire)	Weight gain between birth and 1 year was not associated with PA score (P = 0.95).	Sex, birth weight (for infant growth), infant feeding.
	**Van Deutekom, 2015**[54]	Prospective cohort	N = 194, 54% male, the Netherlands	8.7y	Physical activity (accelerometry)	Weight gain between birth and 12 months was not related to time in MVPA (β = -1.12 min*day^-1^*ΔSD^-1^; 95%-CI: -3.93; 1.69).	GA, Sex, age, SES, parental height and BMI, breast feeding, smoking during pregnancy.
Sedentary behavior							
Determinant	**Author, publication year**	**Type of study**	**Population descriptives (n, % male, country)**	**Mean age at time of outcome assessment**	**Outcome (type and method of measurement)**	**Relevant result**	**Adjustment for confounders**
Normal birth weight	**Gopinath, 2013**[14]	Prospective cohort	N = 1,794, 49% male, Australia. Resurvey at 17–18y: n = 1,213	12.7y	Screen time (questionnaire)	Birth weight (ranked and grouped in quartiles) was not associated with screen time (P_trend_ = 0.77 at 12 year. P_trend_ = 0.48 at 17–18y).	GA, sex, age, ethnicity, BMI, parental education, home ownership, exposure to passive smoking.
	**Pearce, 2012**[51]	Prospective cohort	N = 339, 50% male, United Kingdom	8–10y	SB (accelerometry)	There was no significant association between standardized birth weight and SB (r = 0.016, P>0.05).	Sex, season of measurement.
	**Said-Mohamed, 2012**[52]	Retrospective cohort	N = 162, 56% male, Cameroon	4.1y	SB (accelerometry)	Birth-weight is not correlated with time spent in minimal and sedentary activities (data not shown).	Sex, age, body composition.
	**Hildebrand, 2015**[47]	Meta-analysis of eight cohorts	N = 10,793, 47% male, 6 European countries and Brazil	11.5y	SB (accelerometry)	Birth weight was positively associated with mean daily sedentary time (β = 4.04 min*kg^-1^; 95%-CI: 1.14; 6.94).	Sex, age, study, monitor wear time.
	**Van Deutekom, 2015**[54]	Prospective cohort	N = 194, 54% male, the Netherlands	8.7y	SB (accelerometry)	Birth weight was positively associated with sedentary time (β = 9.88 min*day^-1^*SD^-1^; 95%-CI: 0.74; 19.01).	GA, Sex, age, SES, parental height and BMI, breast feeding, smoking during pregnancy.
	**Wijtzes, 2013**[55]	Prospective cohort	N = 347, 52.4% male, the Netherlands	2.1y	SB (accelerometry)	Birth weight <2,500g was not associated with percentage of time spent in SB, compared to birth weight >2,500g (difference: 2.4%, 95%-CI: -0.4; 5.1).	GA, sex, age, motor development, season of measurement, breast feeding maternal BMI, number of siblings, daycare attendance, household income.
Extreme birth weight	**Kaseva, 2015**[57]	Prospective cohort*	VLBW adults, N = 57 vs. N = 47 controls, 36% male, Finland	24.7y	SB (accelerometry)	Between VLBW and NBW adults, there was no difference in daily sedentary time (mean difference: 14.1 c.p.m.; 95%-CI: -40.4; 68.5).	Sex, age, BMI, season of measurement, smoking, parental education.
	**Said-Mohamed, 2012**[52]	Retrospective cohort	N = 162, 56% male, Cameroon	4.1y	SB (accelerometry)	In a subgroup of children with birth weight <2,5 kg (n = 10), birth-weight is negatively correlated with time spent in minimal and sedentary activities (r = -0.7, P = 0.04).	Sex, age, body composition.
Other birth size	**Gopinath, 2013**[14]	Prospective cohort	N = 1,794, 49% male, Australia. Resurvey at 17–18y: n = 1,213	12.7y	Screen time (questionnaire)	There were no significant associations of either birth length or head circumference with screen time. (data not shown)	GA, sex, age, ethnicity, BMI, parental education, home ownership, exposure to passive smoking.
Infant growth	**Van Deutekom, 2015**[54]	Prospective cohort	N = 194, 54% male, the Netherlands	8.7y	SB (accelerometry)	Infant weight gain was not associated with SB (β = 9.30 min*day^-1^*ΔSD^-1^; -0.58; 19.18).	GA, Sex, age, SES, parental height and BMI, breast feeding, smoking during pregnancy.

Description of the study characteristics, study population, type and measurement of behavior, relevant results and confounders results were adjusted for, sorted by energy balance-related behavior and determinant.

* Same cohort (Helsinki Study of VLBW adults).

^†^ Same cohort.

Abbreviations: BW—Birth weight; BWR—Birth weight ratio; BMI—Body Mass Index; LBW—Low birth weight; NBW—Normal birth weight; HBW—High birth weight; MET—Metabolic Equivalent Task; PA—Physical activity; SGA—Small for gestational age (birth weight < -2 SD); AGA—Appropriate for gestational age; LGA—Large for gestational age (birth weight > +2 SD); PI—Ponderal Index; GA—Gestational age; SES—Socio-economic status; VLBW—Very low birth weight (<1500g); MVPA—Moderate-to-vigorous physical activity; ELBW—Extremely low birth weight (≤800g[58] or <1000g[12]); IUGR—Intrauterine growth retardation; c.p.m.—counts per minute; SB—Sedentary behavior.

### Study characteristics

Thirty-one (76%) of the 41 studies were prospective cohort studies, nine were retrospective studies and one study combined prospective and retrospective cohorts in a meta-analysis.[45] Four articles assessed energy balance-related behavior in the Helsinki Study of Very Low Birth Weight Adults cohort, of which two articles reported self-reported PA level[13, 56], one reported PA assessed by accelerometry[57] and one reported energy intake.[61] Two articles reported data from the Generation R study. [40, 55] Two articles reported eating behavior in the same cohort of children but at a different age.[41, 44] Five articles combined data of several cohorts[11, 43, 45, 47, 53], two of which described pooled data from the International Children’s Accelerometry Database.[11, 47] Thus, we identified data from 35 unique study samples.

The sample size ranged from 84[58] to 43,482 subjects[45], with mean age of the study population ranging from a few days[31] to 70 years.[15] One study included only male subjects[59], and two studies only female subjects.[32, 34] Seven publications reported clinical or otherwise selected populations, namely children with short stature after born small-for-gestational age[38], children with birth weight <1,000g[12] or ≤800g[58] and adults with birth weight <1,500g.[13, 56, 57, 61]

In 39 (95%) of the included studies, birth weight was the primary determinant, expressed as absolute weight, birth weight ratio (i.e., measured birth weight divided by median gestational age adjusted birth weight) or gestational age-adjusted SD-score. Five studies additionally reported other birth size measures besides birth weight (ponderal index, birth length, head circumference, or a combination of these)[14, 15, 33, 48, 49], and one study reported ponderal indices only.[59] Four articles additionally reported the effects of infant growth, besides birth weight.[28, 36, 54, 62] One study addressed only infant growth as primary determinant.[60]

Eight studies reported energy intake as only primary outcome[28, 29, 31, 33, 35, 37, 38, 61], three reported energy intake and PA level[30, 32, 34], six reported eating behavior[39–44], 17 reported only PA levels, seven reported both PA levels and SB[11, 14, 51, 52, 54, 55, 57], and one study reported only SB.[47] In 14 of the studies addressing PA and/or SB, the outcome was parent- or self-reported either by questionnaire or interview. In the remainder, PA was objectively assessed by accelerometry.

Thirty-one (76%) of the 41 studies adjusted for a variety of covariates in the analysis. Eleven studies controlled for confounding by gestational age, which was the criterion for moderate quality in the dimension ‘potential confounding’.

### Quality assessment

The methodological quality of the included studies is presented in Table 3. The scoring of the 41 studies led to an initial disagreement in 37 of a total of 205 domains (18%). Disagreement was mainly prevalent in the ‘study attrition’ domain, because attrition rates were frequently unclearly reported or only mentioned in other publications referred to by the authors. The two reviewers reached consensus on all initial disagreements. Three studies were judged to be of high overall quality[48, 60, 62], 11 studies were of moderate quality, and the remainder of 27 studies was judged to be low overall quality. The most common weakness was the absence of a clear description of the psychometric properties of the measures and not controlling for relevant confounding variables in the analyses.

**Table 3:** Quality assessment of the included studies.

Author, year, reference	Selection bias	Confounding	Measurement	Study attrition	Data analysis	Overall quality	Comment
*High-quality studies*							
Hallal, 2006[62]	◉	●	●	●	●	●	
Hallal, 2012[60]	◉	●	●	●	●	●	
Kehoe, 2002[48]	◉	●	●	◉	●	●	
*Medium-quality studies*							
Atladottir, 2000[28]	◉	○	◉	◉	◉	◉	
Barbieri, 2009[29]	●	◉	○	◉	●	◉	
Cardona Cano, 2015 [40]	◉	◉	◉	○	●	◉	
Gopinath, 2013[14]	◉	●	○	◉	●	◉	
Migraine, 2013[42]	○	◉	◉	●	●	◉	
Pearce, 2012[51]	○	●	●	◉	●	◉	
Perälä, 2012[33]	◉	●	○	●	●	◉	
Ridgway, 2011[11]	◉	●	●	○	●	◉	
Silveira, 2012[44]	◉	◉	◉	◉	●	◉	Identical study population as Escobar, 2014
Van Deutekom, 2015[54]	○	●	●	●	●	◉	
Wijtzes, 2013[55]	◉	●	●	○	●	◉	
*Low-quality studies*							
Andersen, 2009[45]	◉	●	○	○	●	○	
Boone-Heinonen, 2015[30]	◉	○	○	◉	●	○	
Boonstra, 2006[38]	○	○	○	●	○	○	
Brown, 2012[39]	○	○	○	○	○	○	
Campbell, 2010[46]	◉	○	●	○	◉	○	
Davies, 2006[10]	○	○	○	○	◉	○	
Dubignon, 1969[31]	◉	○	●	●	○	○	
Eriksson, 2004[15]	◉	◉	○	○	◉	○	
Escobar, 2014[41]	◉	◉	○	○	●	○	Identical study population as Silveira, 2012
Hack, 2012[12]	◉	○	○	●	○	○	
Hildebrand, 2015[47]	◉	○	●	○	●	○	
Kajantie, 2010[56]	◉	○	○	◉	●	○	Identical study population as Kaseva, 2012, Kaseva, 2013 and Kaseva, 2015
Kaseva, 2012[13]	◉	○	○	◉	●	○	Identical study population as Kajantie, 2010, Kaseva, 2013 and Kaseva, 2015
Kaseva, 2013[61]	◉	○	○	●	◉	○	Identical study population as Kajantie, 2010, Kaseva, 2012 and Kaseva, 2015
Kaseva, 2015[57]	◉	○	●	○	●	○	Identical study population as Kajantie, 2010, Kaseva, 2012 and Kaseva, 2013
Laaksonen, 2003[59]	○	○	○	○	◉	○	
Li, 2015[32]	◉	○	○	●	◉	○	
Mattocks, 2008[49]	○	○	●	●	●	○	
Oliveira, 2015[43]	◉	◉	○	○	●	○	
Ounsted, 1975[37]	○	○	○	○	○	○	
Pahkala, 2010[50]	○	○	○	○	○	○	
Robinson, 2013[36]	○	○	○	●	●	○	
Rogers, 2005[58]	◉	○	○	◉	◉	○	
Ruiz-Narváez, 2014[34]	○	○	○	●	◉	○	
Said-Mohamed, 2012[52]	◉	○	●	○	●	○	
Salbe, 1998[53]	○	○	●	○	◉	○	
Shultis, 2005[35]	●	●	○	○	●	○	

Results of the quality assessment of the included studies, with each dimension judged as strong(●), moderate (◉) or weak (○) based on the judgment rules as defined in Table 1.

### Associations of birth weight and infant growth with energy balance-related behavior

**Fig 2:**
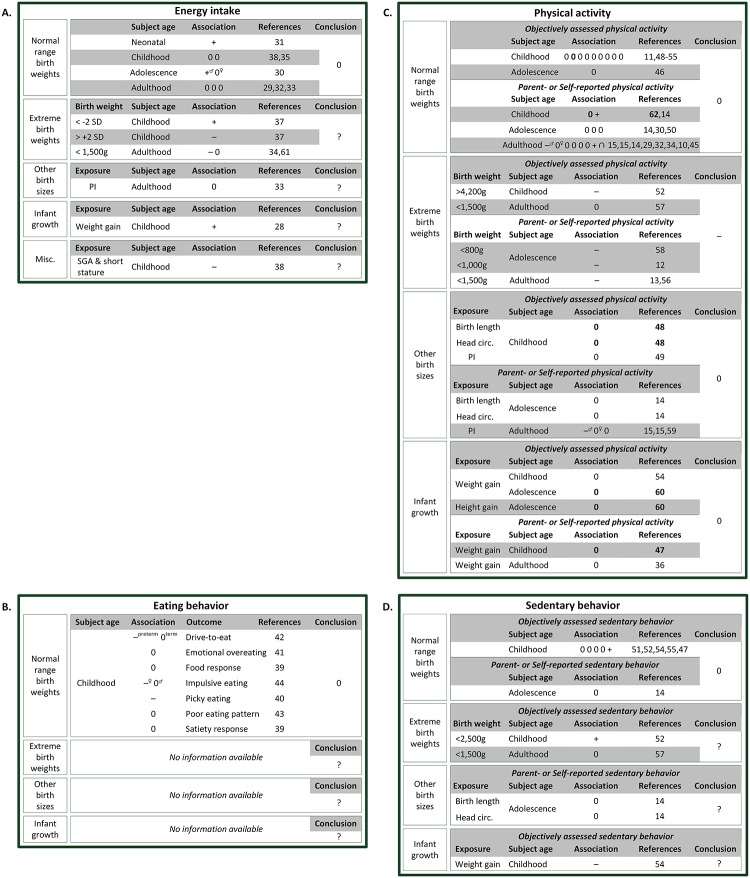
Schematic overview of all the available evidence of birth weight and infant growth with energy intake (A), eating behavior (B), physical activity (C) and sedentary behavior (D) in humans described in the literature to date. Each association is represented by + for positive,—for negative, and 0 for no association. ∩ represents an inversed U-shape association. High quality studies are marked in bold. If the association was only present for a subgroup, the subgroup is specified in superscript. The associations are subdivided by subject’s age: neonatal (age range: 0–1 mo), childhood (1 mo-12yo), adolescence (12–17 yo) and adulthood (18+ yo). The last column lists the composite score of the best-evidence synthesis: + for positive association,—for negative, 0 for no association and? for insufficient evidence. Abbreviations: SGA—Small for gestational age; circ.–circumference; PI—Ponderal Index.

The results for the various associations of birth weight and infant growth with energy balance-related behavior are summarized in Fig 2. Because there is some evidence of an age modification of the association of early growth with energy balance-related behavior, with associations only apparent in adult subjects[45], we present the associations stratified by age. Also, we anticipated a possible difference in association between studies with objectively assessed behaviors and studies with self-/parent-reported behaviors, so we present the associations separately for each method of measurement.

#### Energy intake (Fig 2A)

According to the best-evidence synthesis, we found no evidence for an association of birth weight with energy intake. This is based on three moderate-quality studies[28, 29, 33] and five low-quality studies[30–33, 35], of which only one low-quality study found a positive association of birth weight with energy intake.[31] Also, the evidence for the association of extreme birth weights with energy intake was inconsistent, as the association of low birth weight was absent in one study[61], positive in another[37] and negative in a third.[34] We found insufficient evidence for an association of other birth sizes or infant growth with energy intake in humans.

#### Eating behavior (Fig 2B)

We found no evidence for an association of birth weight with eating behaviors, based on two moderate-quality studies[40, 42] and four low-quality studies.[39, 41, 43, 44] No studies assessed the association of any other of the determinants of interest with eating behavior, resulting in insufficient evidence.

#### Physical activity (Fig 2C)

No evidence was found for an association between birth weight and PA in humans. This is based on the results of two studies of high quality[48, 62], five studies of moderate quality[11, 14, 29, 51, 54] and 12 studies of low quality. Fourteen of the 19 studies, including both high-quality studies and all of the studies objectively assessing PA, found no significant association of birth weight with PA. There was also no evidence for an association of other birth sizes[14, 15, 48, 49, 59] or infant growth[36, 54, 60, 62] with later PA. However, four out of five low-quality studies focusing on extreme birth weights found lower PA levels in very high and very low birth weight individuals[12, 52, 56–58], resulting in moderate evidence for an association of extreme birth weights at both ends of the spectrum with lower PA.

#### Sedentary behavior (Fig 2D)

There is no evidence for an association of birth weight with SB in humans, based on three moderate-quality and two low-quality studies.[14, 47, 51, 52, 55] However, the one study reporting a positive association of birth weight with SB had a sample size of more than 10,000 subjects[47] compared to 2,642 subjects in the other four studies combined.

There is inconsistent evidence for the association of extreme birth weights with SB, as the association with low birth weight was absent in one study[57] and positive in another.[52] The evidence for the association of other birth sizes and infant growth with SB was insufficient.

## Discussion

This review represents the first synthesis of all the available human data on the association of birth weight and infant growth with the spectrum of behaviors that collectively encompass energy balance-related behavior, while accounting for the methodological quality of the studies. We identified 41 eligible studies, of which the large majority focused on the association of birth weight with PA. Overall, there is no evidence for an association of birth weight with PA, SB, energy intake or eating behaviors, although the largest study addressing SB found that a kilogram increase in birth weight was associated with 4 more minutes of daily SB.[47] There was no indication that age modified the association of birth weight with the different outcome measures. The results based on studies with objectively assessed behaviors versus studies with self-/parent-reported behaviors were similar.

Despite evidence supporting the idea that postnatal weight gain is more important than birth weight for later obesity risk[64], the human evidence on the association of infant growth with energy balance-related behaviors is generally insufficient. Four low- and medium-quality studies on infant growth and later PA levels found no evidence for an association, but insufficient evidence was present on the association of infant growth with energy intake, eating behavior or SB.

We found moderate evidence for the association of extreme birth weights with later PA levels, as studies in very low birth weight[13, 56] and extremely low birth weight subjects[12, 58], and a subgroup analysis in subjects with birth weights >4,200g[52], showed lower PA levels in these subjects compared to normal birth weight controls. At present these studies do not allow firm conclusions, due to the paucity of studies and variations in applied birth weight cut-off points. In addition, the extrapolation from conclusions based on subjects with extreme birth weights to the normal birth weight spectrum cannot be done. Prenatal conditions leading to extreme birth weights may have teratogenic or disruptive effects, and the associated comorbidities could lead to poorer motor performance, neurosensory impairments or parental overprotection, which could hamper subjects in their PA participation.[56] The relevance of these disruptive consequences to the origins of energy balance-related behavior in the general population seems remote.

We conducted a best-evidence synthesis to summarize all the available evidence on the association of pre- and early postnatal growth with energy balance related behavior. This has the additional advantage that it accounts for the methodological quality of the studies: low- and moderate-quality studies are disregarded if sufficient high-quality evidence is available. However, this approach does not consider the size of the studies involved. Many of the studies were relatively small, particularly if samples were stratified by age or sex. For example, in the study of Salbe *et al*. the relatively small sample size of 88 children may have compromised the study’s ability to detect an association of birth weight with PA.[53] But in the best-evidence synthesis it carries an equal weight to an individual level meta-analysis of more than 43,000 adult subjects.[45] This latter study found a small but significant inversed U-shaped association between birth weight and PA levels, with odds ratios of being active of 0.67 and 0.65 in the lowest and highest birth weight subjects, respectively, compared to normal birth weight subjects. In another large study of over 10,000 youths, a kilogram higher birth weight was associated with four more minutes of daily SB per kilogram increase in birth weight, which approximates to 1% of mean daily sedentary time.[47] The clinical relevance of these small perturbations in energy balance-related behavior across the normal birth weight range is debatable, but future studies should be of sufficient power to detect small but relevant effect sizes with sufficient confidence. A practical solution may be to combine existing cohort studies in which both simple growth indices and detailed outcome measures have been assessed. These individual level meta-analyses, such as done in the NordNet study[45] and the International Children’s Accelerometry Database[47], have been productive in epidemiological research on the developmental origins of obesity to date, and represent a potentially valuable existing resource for future studies on the underlying pathways.

A second limitation of the best-evidence synthesis is that it does not give an estimated overall effect size. For this, a meta-analysis would have been preferable, but this requests statistical pooling. Pooling was only possible for the continuous association of birth weight expressed in kg with PA expressed in accelerometer counts per minute, which was reported in three publications.[11, 48, 49] Øglund *et al*. previously reviewed the literature on the association of birth weight with PA, but their review was limited to studies in children and to studies with PA assessed by accelerometry.[22] They conducted a meta-analysis including these three studies, complemented with unpublished data from two other studies.[46, 55] This meta-analysis of five publications encompassing 18,602 subjects resulted in an overall mean effect size of -3.08 accelerometer c.p.m. per kilogram birth weight (95%CI: -10.2; 4.04), suggesting that there is a very small, non-significant inverse association of birth weight with PA in youth. By another approach this leads to the same conclusion of our best-evidence synthesis of 18 studies, i.e., that there is no evidence for an association of birth weight with PA.

As with any systematic review, this review is limited by the quality of the included studies. The quality of the included studies was generally low, with the exception of 11 studies judged to be of moderate quality, and three studies of high quality. The most common shortcoming in the methodological quality of the included studies was that the required information to assess the validity and reliability of the behaviors was often lacking or unclearly reported. Reliability and validity of the data may be of particular concern in studies where data were based on recall or self-report albeit using validated instruments.[65] These measurements are prone to misclassification, due to aspects of social desirability and recall bias. We encourage the use of more reliable and valid measures, e.g. accelerometry for the assessment of PA and SB. In addition, more than 40% of the studies scored weak on the quality dimension ‘attrition’, signaling that the follow-up rate was less than 60% or the drop-outs were not described. A low attrition rate might lead to an overestimation of an association if loss to follow up was differential, for example if VLBW subjects who are limited in their exercise capacity would be more prone to adhere than unimpaired subjects.[56]

Systematic reviews are subject to publication bias. Publication bias typically biases the association away from null, so this might particular be relevant for the (positive) association of extreme birth weights with PA.

Our review was restricted to the association of birth weight and postnatal growth with energy balance-related behaviors as primary outcomes. Therefore, studies focusing on other potentially relevant factors such as basal metabolic rate, its regulatory systems, such as the autonomic nervous system, or closely related pathways, such as the timing of the ‘obesity rebound’. Birth weight has been associated with resting metabolic rate[66], and infant weight gain with autonomic nervous system activity.[67] The age of the ‘adiposity rebound’ (the rise in childhood BMI, after an initial drop, that occurs between age 3 and 6) is considered of critical importance to the setting of energy balance, and empirical evidence suggests that suboptimal perinatal growth advances the timing of adiposity rebound.[68, 69] All these factors have been suggested to contribute to an elevated risk of cardiovascular disease and obesity, and could therefore represent other potential underlying pathways from early growth to later obesity. In addition, we excluded studies relating major gestational food restriction to energy balance-related behaviors. These studies reported that prenatal exposure to the Dutch famine is associated with increased energy intake[70] and less sports participation[71] at middle age. However, these subjects were exposed to environmental stress (war), besides malnutrition, which may induce additional developmental changes. Both prenatal stress and nutritional deprivation are not necessarily accompanied by a reduction in birth weight or change in body composition in early life. Therefore, famine exposure is a unique event, not easily comparable to low birth weight and not generalizable to a suboptimal prenatal environment at present. Therefore, we excluded these studies from the present review.

In conclusion, the studies included in our best-evidence synthesis indicate that there is no evidence for an association of birth weight with PA, SB, energy intake or eating behaviors. Also, there is no evidence that other birth sizes or infant growth are associated with PA in later life. However, there is moderate evidence for an association of extreme birth weights with lower PA. There is insufficient evidence on the associations of infant growth, other birth size measures and extreme birth sizes with energy intake, eating behavior or SB.

Our study leads to important recommendations for future research. First, as a relatively high number of studies found no association of birth weight with any energy balance-related behavior, shifting focus to the effects of infant growth or trimester specific fetal growth[72] on these behaviors might be more fruitful. Second, the association of early growth with energy balance in a broader context, e.g. including basal metabolic rate and adipogenesis, was beyond the scope of this review but will potentially add to the ability to explain the developmental origins of obesity. Third, pooling of data that permit individual level meta-analyses would help ensure sufficient power to detect small perturbations in energy balance.

Obesity prevention and treatment programs may be helped with better identification of mechanisms that underlie relationships between early life growth and adult obesity, but current evidence does not allow inferences about the relation of early-life growth with energy balance-related behavior in later life. First, there is a need of high-quality studies on this topic that overcome the methodological limitations in participation, measurements and attrition rate that almost invariably accompany existing birth cohorts.

## Supporting Information

S1 File PRISMA 2009 checklist. (DOC)

S2 File Full literature search strategy and number of references. (PDF)

## Abbreviations

PA: Physical Activity
SB: Sedentary Behavior
