# Supplementary material for: The Association of Birth Weight and Infant Growth with Energy Balance-Related Behavior – A Systematic Review and Best-Evidence Synthesis of Human Studies
Source: PLoS One. 2017 Jan 12;12(1):e0168186. doi: 10.1371/journal.pone.0168186 (PMC5232347; doi:10.1371/journal.pone.0168186)
Supplement: S2 File — (PDF) [file pone.0168186.s002.pdf]

Online Supplementary Material to the article:

The Association of Birth weight and Infant growth with Energy Balance-Related Behavior – A Systematic Review and Best-evidence Synthesis of Human Studies

Arend W. van Deutekom[1]; Mai J.M. Chinapaw[2]; E. (Ilse) P. Jansma[3]; Tanja G.M. Vrijkotte[4]; Reinoud J.B.J. Gemke[1]

[1] Department of Pediatrics, EMGO Institute for Health & Care Research, Institute for Cardiovascular Research VU, VU University Medical Center, Amsterdam, the Netherlands; [2] Department of Public and Occupational Health, EMGO institute for Health & Care Research, VU University Medical Center, Amsterdam, the Netherlands; [3] Department of Epidemiology and Biostatistics, EMGO institute for Health & Care Research, VU University Medical Centre, Amsterdam, Netherlands; [4] Department of Public Health, Academic Medical Centre, University of Amsterdam, Amsterdam, the Netherlands.

Full literature search and number of references:

**Search date**	: 15-01-2013	update 03-07-2014	update 19-05-2015
Pubmed	: 2898	3275	3514
Embase	: 2356	3773	2879
PsycInfo	: 490	527	548
Cochrane	: 705	402	482
Total	: 6449	7977	7423
After deduplication	: 5530	6853	6231

Search date	15-1-2013	Update 3-7-2014	Update 19-5-2015	Update 5-1-2016
Pubmed	2898	3275	3514	3724
Embase	2356	3773	2879	3061
PsycInfo	490	527	548	577
Cochrane	705	402	482	545
**Total**	6369	7977	7423	7907
**After deduplication**	5530	6853	6231	6688

Methods: Liberati et al., The PRISMA statement for reporting systematic reviews and meta-analyses of studies that evaluate helath care interventions: explanation and elaboration. 2009, Journal of Clinical Epidemiology; 62: e1-e34.

1. Pubmed

#1 Perinatal influences
Birth weight[Mesh] OR "Infant, Low Birth Weight"[Mesh] OR "Prenatal Exposure Delayed Effects"[Mesh] OR dohad[tiab] OR (("Birth weight"[tiab] OR "Perinatal"[tiab] OR "infant growth"[tiab] OR "fetal"[tiab] OR "intra-uterine growth"[tiab] OR "infant programming"[tiab] OR "intra-uterine programming"[tiab] OR "developmental programming"[tiab] OR "intra-uterine plasticity"[tiab] OR "developmental plasticity"[tiab] OR developmental origin*[tiab] OR early-life origin*[tiab] OR intra-uterine origin*[tiab] OR developmental influence*[tiab] OR early-life influence*[tiab] OR intra-uterine influence*[tiab] OR famine[tiab] OR "catch-up growth"[tiab] OR "accelerated growth"[tiab] OR "Small for Gestational Age"[tiab] OR dysmaturity[tiab]) NOT medline[sb])

#2 Energy-balance related behavior
Physical activity - Sedentary behavior - Eating behavior – Energy intake

"Exercise"[Majr:NoExp] OR "Running"[Mesh] OR "Swimming"[Mesh:noexp] OR "Walking"[Mesh:noexp] OR "Sports"[Mesh] OR "Life Style"[Mesh:NoExp] OR "Sedentary Lifestyle"[Mesh] OR "Appetite"[Mesh] OR "Health Behavior"[Majr:NoExp] OR "Feeding Behavior"[Mesh] OR "physical activity"[tiab] OR appetit*[tiab] OR satiety[tiab] OR "childhood diet"[tiab] OR ((sport*[tiab] OR exercise*[tiab] OR "activity level"[tiab] OR "motor activity"[tiab] OR "locomotor activity"[tiab] OR "sedentary"[tiab] OR sedentarism[tiab] OR sedentariness[tiab] OR "physical inactivity"[tiab] OR "sitting"[tiab] OR "diet behavior"[tiab] OR "dietary behavior"[tiab] OR "feeding behavior"[tiab] OR "appetitive behavior"[tiab] OR "diet behaviour"[tiab] OR "dietary behaviour"[tiab] OR "feeding behaviour"[tiab] OR "appetitive behaviour"[tiab] OR diet preference*[tiab] OR dietary preference*[tiab] OR feeding preference*[tiab] OR diet habit*[tiab] OR dietary habit*[tiab] OR feeding habit*[tiab] OR "feeding practice"[tiab] OR "feeding practices"[tiab] OR "diet regulation"[tiab] OR "feeding regulation"[tiab] OR "diet control"[tiab] OR "dietary control"[tiab] OR "feeding control"[tiab] OR hunger[tiab] OR hyperphagia[tiab] OR hyperphagic[tiab] OR "energy expenditure"[tiab] OR "energy level"[tiab]) NOT medline[sb])

#3 Publication types filter:
NOT ("addresses"[Publication Type] OR "biography"[Publication Type] OR "comment"[Publication Type] OR "directory"[Publication Type] OR "editorial"[Publication Type] OR "festschrift"[Publication Type] OR "interview"[Publication Type] OR "lectures"[Publication Type] OR "legal cases"[Publication Type] OR "legislation"[Publication Type] OR "letter"[Publication Type] OR "news"[Publication Type] OR "newspaper article"[Publication Type] OR "patient education handout"[Publication Type] OR "popular works"[Publication Type] OR "congresses"[Publication Type] OR "consensus development conference"[Publication Type] OR "consensus development conference, nih"[Publication Type] OR "practice guideline"[Publication Type])

Search	PubMed Query 15-01-2013	Items found
#6	Search **#4 AND #5**	12
#5	Search **20493499[uid] OR 15997047[uid] OR 23049962[uid] OR 23251693[uid] OR 21167701[uid] OR 16234423[uid] OR 19064527[uid] OR 16404403[uid] OR 16339179[uid] OR 15469656[uid] OR 22595039[uid] OR 18761782[uid]**	12
#4	Search **#3 NOT ("addresses"[Publication Type] OR "biography"[Publication Type] OR "comment"[Publication Type] OR "directory"[Publication Type] OR "editorial"[Publication Type] OR "festschrift"[Publication Type] OR "interview"[Publication Type] OR "lectures"[Publication Type] OR "legal cases"[Publication Type] OR "legislation"[Publication Type] OR "letter"[Publication Type] OR "news"[Publication Type] OR "newspaper article"[Publication Type] OR "patient education handout"[Publication Type] OR "popular works"[Publication Type] OR "congresses"[Publication Type] OR "consensus development conference"[Publication Type] OR "consensus development conference, nih"[Publication Type] OR "practice guideline"[Publication Type])**	2898
#3	Search **#1 AND #2**	3004
#2	Search **"Exercise"[Majr:NoExp] OR "Running"[Mesh] OR "Swimming"[Mesh:noexp] OR "Walking"[Mesh:noexp] OR "Sports"[Mesh] OR "Life Style"[Mesh:NoExp] OR "Sedentary Lifestyle"[Mesh] OR "Appetite"[Mesh] OR "Health Behavior"[Majr:NoExp] OR "Feeding Behavior"[Mesh] OR "physical activity"[tiab] OR appetit*[tiab] OR satiety[tiab] OR "childhood diet"[tiab] OR ((sport*[tiab] OR exercise*[tiab] OR "activity level"[tiab] OR "motor activity"[tiab] OR "locomotor activity"[tiab] OR "sedentary"[tiab] OR sedentarism[tiab] OR sedentariness[tiab] OR "physical inactivity"[tiab] OR "sitting"[tiab] OR "diet behavior"[tiab] OR "dietary behavior"[tiab] OR "feeding behavior"[tiab] OR "appetitive behavior"[tiab] OR "diet behaviour"[tiab] OR "dietary behaviour"[tiab] OR "feeding behaviour"[tiab] OR "appetitive behaviour"[tiab] OR diet preference*[tiab] OR dietary preference*[tiab] OR feeding preference*[tiab] OR diet habit*[tiab] OR dietary habit*[tiab] OR feeding habit*[tiab] OR "feeding practice"[tiab] OR "feeding practices"[tiab] OR "diet regulation"[tiab] OR "feeding regulation"[tiab] OR "diet control"[tiab] OR "dietary control"[tiab] OR "feeding control"[tiab] OR hunger[tiab] OR hyperphagia[tiab] OR hyperphagic[tiab] OR "energy expenditure"[tiab] OR "energy level"[tiab]) NOT medline[sb])**	355277
#1	Search **Birth weight[Mesh] OR "Infant, Low Birth Weight"[Mesh] OR "Prenatal Exposure Delayed Effects"[Mesh] OR dohad[tiab] OR (("Birth weight"[tiab] OR "Perinatal"[tiab] OR "infant growth"[tiab] OR "fetal"[tiab] OR "intra-uterine growth"[tiab] OR "infant programming"[tiab] OR "intra-uterine programming"[tiab] OR "developmental programming"[tiab] OR "intra-uterine plasticity"[tiab] OR "developmental plasticity"[tiab] OR developmental origin*[tiab] OR early-life origin*[tiab] OR intra-uterine origin*[tiab] OR developmental influence*[tiab] OR early-life influence*[tiab] OR intra-uterine influence*[tiab] OR famine[tiab] OR "catch-up growth"[tiab] OR "accelerated growth"[tiab] OR "Small for Gestational Age"[tiab] OR dysmaturity[tiab]) NOT medline[sb])**	80276

Search	PunMed Query 03-07-2014	Items found
#4	Search **#3 NOT ("addresses"[Publication Type] OR "biography"[Publication Type] OR "comment"[Publication Type] OR "directory"[Publication Type] OR "editorial"[Publication Type] OR "festschrift"[Publication Type] OR "interview"[Publication Type] OR "lectures"[Publication Type] OR "legal cases"[Publication Type] OR "legislation"[Publication Type] OR "letter"[Publication Type] OR "news"[Publication Type] OR "newspaper article"[Publication Type] OR "patient education handout"[Publication Type] OR "popular works"[Publication Type] OR "congresses"[Publication Type] OR "consensus development conference"[Publication Type] OR "consensus development conference, nih"[Publication Type] OR "practice guideline"[Publication Type])**	3275
#3	Search **#1 AND #2**	3392
#2	Search **"Exercise"[Majr:NoExp] OR "Running"[Mesh] OR "Swimming"[Mesh:noexp] OR "Walking"[Mesh:noexp] OR "Sports"[Mesh] OR "Life Style"[Mesh:NoExp] OR "Sedentary Lifestyle"[Mesh] OR "Appetite"[Mesh] OR "Health Behavior"[Majr:NoExp] OR "Feeding Behavior"[Mesh] OR "physical activity"[tiab] OR appetit*[tiab] OR satiety[tiab] OR "childhood diet"[tiab] OR ((sport*[tiab] OR exercise*[tiab] OR "activity level"[tiab] OR "motor activity"[tiab] OR "locomotor activity"[tiab] OR "sedentary"[tiab] OR sedentarism[tiab] OR sedentariness[tiab] OR "physical inactivity"[tiab] OR "sitting"[tiab] OR "diet behavior"[tiab] OR "dietary behavior"[tiab] OR "feeding behavior"[tiab] OR "appetitive behavior"[tiab] OR "diet behaviour"[tiab] OR "dietary behaviour"[tiab] OR "feeding behaviour"[tiab] OR "appetitive behaviour"[tiab] OR diet preference*[tiab] OR dietary preference*[tiab] OR feeding preference*[tiab] OR diet habit*[tiab] OR dietary habit*[tiab] OR feeding habit*[tiab] OR "feeding practice"[tiab] OR "feeding practices"[tiab] OR "diet regulation"[tiab] OR "feeding regulation"[tiab] OR "diet control"[tiab] OR "dietary control"[tiab] OR "feeding control"[tiab] OR hunger[tiab] OR hyperphagia[tiab] OR hyperphagic[tiab] OR "energy expenditure"[tiab] OR "energy level"[tiab]) NOT medline[sb])**	408780
#1	Search **Birth weight[Mesh] OR "Infant, Low Birth Weight"[Mesh] OR "Prenatal Exposure Delayed Effects"[Mesh] OR dohad[tiab] OR (("Birth weight"[tiab] OR "Perinatal"[tiab] OR "infant growth"[tiab] OR "fetal"[tiab] OR "intra-uterine growth"[tiab] OR "infant programming"[tiab] OR "intra-uterine programming"[tiab] OR "developmental programming"[tiab] OR "intra-uterine plasticity"[tiab] OR "developmental plasticity"[tiab] OR developmental origin*[tiab] OR early-life origin*[tiab] OR intra-uterine origin*[tiab] OR developmental influence*[tiab] OR early-life influence*[tiab] OR intra-uterine influence*[tiab] OR famine[tiab] OR "catch-up growth"[tiab] OR "accelerated growth"[tiab] OR "Small for Gestational Age"[tiab] OR dysmaturity[tiab]) NOT medline[sb])**	89121

Search	PubMed Query 19-05-2015	Items found
#4	Search **#3 NOT ("addresses"[Publication Type] OR "biography"[Publication Type] OR "comment"[Publication Type] OR "directory"[Publication Type] OR "editorial"[Publication Type] OR "festschrift"[Publication Type] OR "interview"[Publication Type] OR "lectures"[Publication Type] OR "legal cases"[Publication Type] OR "legislation"[Publication Type] OR "letter"[Publication Type] OR "news"[Publication Type] OR "newspaper article"[Publication Type] OR "patient education handout"[Publication Type] OR "popular works"[Publication Type] OR "congresses"[Publication Type] OR "consensus development conference"[Publication Type] OR "consensus development conference, nih"[Publication Type] OR "practice guideline"[Publication Type])**	3514
#3	Search **#1 AND #2**	3640
#2	Search **"Exercise"[Majr:NoExp] OR "Running"[Mesh] OR "Swimming"[Mesh:noexp] OR "Walking"[Mesh:noexp] OR "Sports"[Mesh] OR "Life Style"[Mesh:NoExp] OR "Sedentary Lifestyle"[Mesh] OR "Appetite"[Mesh] OR "Health Behavior"[Majr:NoExp] OR "Feeding Behavior"[Mesh] OR "physical activity"[tiab] OR appetit*[tiab] OR satiety[tiab] OR "childhood diet"[tiab] OR ((sport*[tiab] OR exercise*[tiab] OR "activity level"[tiab] OR "motor activity"[tiab] OR "locomotor activity"[tiab] OR "sedentary"[tiab] OR sedentarism[tiab] OR sedentariness[tiab] OR "physical inactivity"[tiab] OR "sitting"[tiab] OR "diet behavior"[tiab] OR "dietary behavior"[tiab] OR "feeding behavior"[tiab] OR "appetitive behavior"[tiab] OR "diet behaviour"[tiab] OR "dietary behaviour"[tiab] OR "feeding behaviour"[tiab] OR "appetitive behaviour"[tiab] OR diet preference*[tiab] OR dietary preference*[tiab] OR feeding preference*[tiab] OR diet habit*[tiab] OR dietary habit*[tiab] OR feeding habit*[tiab] OR "feeding practice"[tiab] OR "feeding practices"[tiab] OR "diet regulation"[tiab] OR "feeding regulation"[tiab] OR "diet control"[tiab] OR "dietary control"[tiab] OR "feeding control"[tiab] OR hunger[tiab] OR hyperphagia[tiab] OR hyperphagic[tiab] OR "energy expenditure"[tiab] OR "energy level"[tiab]) NOT medline[sb])**	436156
#1	Search **Birth weight[Mesh] OR "Infant, Low Birth Weight"[Mesh] OR "Prenatal Exposure Delayed Effects"[Mesh] OR dohad[tiab] OR (("Birth weight"[tiab] OR "Perinatal"[tiab] OR "infant growth"[tiab] OR "fetal"[tiab] OR "intra-uterine growth"[tiab] OR "infant programming"[tiab] OR "intra-uterine programming"[tiab] OR "developmental programming"[tiab] OR "intra-uterine plasticity"[tiab] OR "developmental plasticity"[tiab] OR developmental origin*[tiab] OR early-life origin*[tiab] OR intra-uterine origin*[tiab] OR developmental influence*[tiab] OR early-life influence*[tiab] OR intra-uterine influence*[tiab] OR famine[tiab] OR "catch-up growth"[tiab] OR "accelerated growth"[tiab] OR "Small for Gestational Age"[tiab] OR dysmaturity[tiab]) NOT medline[sb])**	94299

Search	PubMed Query 05-01-2016	Items found
#52	Search **#51 NOT ("addresses"[Publication Type] OR "biography"[Publication Type] OR "comment"[Publication Type] OR "directory"[Publication Type] OR "editorial"[Publication Type] OR "festschrift"[Publication Type] OR "interview"[Publication Type] OR "lectures"[Publication Type] OR "legal cases"[Publication Type] OR "legislation"[Publication Type] OR "letter"[Publication Type] OR "news"[Publication Type] OR "newspaper article"[Publication Type] OR "patient education handout"[Publication Type] OR "popular works"[Publication Type] OR "congresses"[Publication Type] OR "consensus development conference"[Publication Type] OR "consensus development conference, nih"[Publication Type] OR "practice guideline"[Publication Type])**	3724
#51	Search **#49 AND #50**	3855
#50	Search **"Exercise"[Majr:NoExp] OR "Running"[Mesh] OR "Swimming"[Mesh:noexp] OR "Walking"[Mesh:noexp] OR "Sports"[Mesh] OR "Life Style"[Mesh:NoExp] OR "Sedentary Lifestyle"[Mesh] OR "Appetite"[Mesh] OR "Health Behavior"[Majr:NoExp] OR "Feeding Behavior"[Mesh] OR "physical activity"[tiab] OR appetit*[tiab] OR satiety[tiab] OR "childhood diet"[tiab] OR ((sport*[tiab] OR exercise*[tiab] OR "activity level"[tiab] OR "motor activity"[tiab] OR "locomotor activity"[tiab] OR "sedentary"[tiab] OR sedentarism[tiab] OR sedentariness[tiab] OR "physical inactivity"[tiab] OR "sitting"[tiab] OR "diet behavior"[tiab] OR "dietary behavior"[tiab] OR "feeding behavior"[tiab] OR "appetitive behavior"[tiab] OR "diet behaviour"[tiab] OR "dietary behaviour"[tiab] OR "feeding behaviour"[tiab] OR "appetitive behaviour"[tiab] OR diet preference*[tiab] OR dietary preference*[tiab] OR feeding preference*[tiab] OR diet habit*[tiab] OR dietary habit*[tiab] OR feeding habit*[tiab] OR "feeding practice"[tiab] OR "feeding practices"[tiab] OR "diet regulation"[tiab] OR "feeding regulation"[tiab] OR "diet control"[tiab] OR "dietary control"[tiab] OR "feeding control"[tiab] OR hunger[tiab] OR hyperphagia[tiab] OR hyperphagic[tiab] OR "energy expenditure"[tiab] OR "energy level"[tiab]) NOT medline[sb])**	467128
#49	Search **Birth weight[Mesh] OR "Infant, Low Birth Weight"[Mesh] OR "Prenatal Exposure Delayed Effects"[Mesh] OR dohad[tiab] OR (("Birth weight"[tiab] OR "Perinatal"[tiab] OR "infant growth"[tiab] OR "fetal"[tiab] OR "intra-uterine growth"[tiab] OR "infant programming"[tiab] OR "intra-uterine programming"[tiab] OR "developmental programming"[tiab] OR "intra-uterine plasticity"[tiab] OR "developmental plasticity"[tiab] OR developmental origin*[tiab] OR early-life origin*[tiab] OR intra-uterine origin*[tiab] OR developmental influence*[tiab] OR early-life influence*[tiab] OR intra-uterine influence*[tiab] OR famine[tiab] OR "catch-up growth"[tiab] OR "accelerated growth"[tiab] OR "Small for Gestational Age"[tiab] OR dysmaturity[tiab]) NOT medline[sb])**	98722

2. EMBASE.com

#1 Perinatal influences/ DOHaD
'birth weight'/exp OR 'prenatal exposure'/exp OR dohad:ti,ab OR 'Perinatal growth':ti,ab OR 'infant growth':ti,ab OR 'intra-uterine growth':ti,ab OR 'intra-uterine programming':ti,ab OR 'developmental programming':ti,ab OR 'developmental origin':ti,ab OR 'developmental origins':ti,ab OR 'early-life origin':ti,ab OR 'early-life origins':ti,ab OR 'catch-up growth':ti,ab OR 'accelerated growth':ti,ab OR 'Small for Gestational Age':ti,ab OR dysmaturity:ti,ab

#2 Energy-balance related behavior
Physical acticity - Sedentary behavior - Eating behaviour

'exercise'/mj OR 'physical activity'/exp OR 'laziness'/exp OR 'lifestyle'/exp OR 'sedentary lifestyle'/exp OR 'feeding behavior'/exp OR 'health behavior'/mj OR 'childhood diet':ti,ab OR hunger:ti,ab OR hyperphagia:ti,ab OR hyperphagic:ti,ab OR 'energy expenditure':ti,ab OR 'energy level':ti,ab

Embase Session Results (15 Jan 2013)

No.	Query	Results
#13	#10 AND #11 AND ([article]/lim OR [article in press]/lim OR [review]/lim OR [short survey]/lim)	2356
#12	#10 AND #11	2915
#11	'exercise'/mj OR 'physical activity'/exp OR 'laziness'/exp OR 'lifestyle'/exp OR 'sedentary lifestyle'/exp OR 'feeding behavior'/exp OR 'health behavior'/mj OR 'childhood diet':ab,ti OR hunger:ab,ti OR hyperphagia:ab,ti OR hyperphagic:ab,ti OR 'energy expenditure':ab,ti OR 'energy level':ab,ti	458090
#10	'birth weight'/exp OR 'prenatal exposure'/exp OR dohad:ab,ti OR 'perinatal growth':ab,ti OR 'infant growth':ab,ti OR 'intra-uterine growth':ab,ti OR 'intra-uterine programming':ab,ti OR 'developmental programming':ab,ti OR 'developmental origin':ab,ti OR 'developmental origins':ab,ti OR 'early-life origin':ab,ti OR 'early-life origins':ab,ti OR 'catch-up growth':ab,ti OR 'small for gestational age':ab,ti OR dysmaturity:ab,ti	89807

Embase Session Results (19 May 2015)

No.	Query	Results
#4	**#3** AND ([article]/lim OR [article in press]/lim OR [review]/lim OR [short survey]/lim)	**2879**
#3	**#1** AND **#2**	**3845**
#2	**'exercise'**/mj OR **'physical activity'**/exp OR **'laziness'**/exp OR **'lifestyle'**/exp OR **'sedentary lifestyle'**/exp OR **'feeding behavior'**/exp OR **'health behavior'**/mj OR **'childhood diet'**:ab,ti OR **hunger**:ab,ti OR **hyperphagia**:ab,ti OR **hyperphagic**:ab,ti OR **'energy expenditure'**:ab,ti OR **'energy level'**:ab,ti	**559006**
#1	**'birth weight'**/exp OR **'prenatal exposure'**/exp OR **dohad**:ab,ti OR **'perinatal growth'**:ab,ti OR **'infant growth'**:ab,ti OR **'intra-uterine growth'**:ab,ti OR **'intra-uterine programming'**:ab,ti OR **'developmental programming'**:ab,ti OR **'developmental origin'**:ab,ti OR **'developmental origins'**:ab,ti OR **'early-life origin'**:ab,ti OR **'early-life origins'**:ab,ti OR **'catch-up growth'**:ab,ti OR **'accelerated growth'**:ab,ti OR **'small for gestational age'**:ab,ti OR **dysmaturity**:ab,ti	**109366**

Embase Session Results (5 Jan 2016)

No.	Query	Results
#4	**#3** AND ([article]/lim OR [article in press]/lim OR [review]/lim OR [short survey]/lim)	**3061**
#3	**#1** AND **#2**	**4094**
#2	**'exercise'**/mj OR **'physical activity'**/exp OR **'laziness'**/exp OR **'lifestyle'**/exp OR **'sedentary lifestyle'**/exp OR **'feeding behavior'**/exp OR **'health behavior'**/mj OR **'childhood diet'**:ab,ti OR **hunger**:ab,ti OR **hyperphagia**:ab,ti OR **hyperphagic**:ab,ti OR **'energy expenditure'**:ab,ti OR **'energy level'**:ab,ti	**589756**
#1	**'birth weight'**/exp OR **'prenatal exposure'**/exp OR **dohad**:ab,ti OR **'perinatal growth'**:ab,ti OR **'infant growth'**:ab,ti OR **'intra-uterine growth'**:ab,ti OR **'intra-uterine programming'**:ab,ti OR **'developmental programming'**:ab,ti OR **'developmental origin'**:ab,ti OR **'developmental origins'**:ab,ti OR **'early-life origin'**:ab,ti OR **'early-life origins'**:ab,ti OR **'catch-up growth'**:ab,ti OR **'accelerated growth'**:ab,ti OR **'small for gestational age'**:ab,ti OR **dysmaturity**:ab,ti	**114861**

3. PsycINFO

#1 Perinatal influences/ DOHaD

DE "Birth Weight" OR DE "Perinatal Period" OR DE "Prenatal Exposure" OR DE "Prenatal Development" OR TI dohad OR AB dohad OR TI "Perinatal growth" OR TI "infant growth" OR TI "intra-uterine growth" OR TI "intra-uterine programming" OR TI "developmental programming" OR TI "developmental origin" OR TI "developmental origins" OR TI "early-life origin" OR TI "early-life origins" OR TI "catch-up growth" OR TI "accelerated growth" OR TI "Small for Gestational Age" OR TI "dysmaturity" OR AB "Perinatal growth" OR AB "infant growth" OR AB "intra-uterine growth" OR AB "intra-uterine programming" OR AB "developmental programming" OR AB "developmental origin" OR AB "developmental origins" OR AB "early-life origin" OR AB "early-life origins" OR AB "catch-up growth" OR AB "accelerated growth" OR AB "Small for GestaABonal Age" OR AB "dysmaturity"

#2 Energy-balance related behavior

Physical acticity - Sedentary behavior - Eating behaviour

DE "Exercise" OR DE "Activity Level" OR DE "Activities of Daily Living" OR DE "Motivation" OR DE "Physical Activity" OR DE "Diets" OR DE "Health Attitudes" OR DE "Lifestyle" OR DE "Active Living" OR DE "Health Behavior" OR DE "Swimming" OR DE "Sports" OR DE "Sports (Attitudes Toward)" OR DE "Motor Performance" OR DE "Jumping" OR DE "Running" OR DE "Walking" OR DE "Eating Behavior" OR DE "Binge Eating" OR DE "Appetite" OR DE "Eating Attitudes" OR DE "Food Intake" OR DE "Mealtimes" OR TI sedentar* OR TI satiety OR TI hunger OR TI hyperphagia OR TI hyperphagic OR TI "energy expenditure" OR TI "energy level" OR AB sedentar* OR AB satiety OR AB hunger OR AB hyperphagia OR AB hyperphagic OR AB "energy expenditure" OR AB "energy level"

#3 Publication types filter:

Limiters - Publication Type: All Journals, Peer Reviewed Journal, Peer-Reviewed Status-Unknown; Document Type: Journal Article
Search modes - Boolean/Phrase

#	PsycINFO Query	Limiters/Expanders	Results
S3	(S1 AND S2)	Limiters - Publication Type: All Journals, Peer Reviewed Journal, Peer-Reviewed Status-Unknown; Document Type: Journal Article Search modes - Boolean/Phrase	493
S2	DE "Exercise" OR DE "Activity Level" OR DE "Activities of Daily Living" OR DE "Motivation" OR DE "Physical Activity" OR DE "Diets" OR DE "Health Attitudes" OR DE "Lifestyle" OR DE "Active Living" OR DE "Health Behavior" OR DE "Swimming" OR DE "Sports" OR DE "Sports (Attitudes Toward)" OR DE "Motor Performance" OR DE "Jumping" OR DE "Running" OR DE "Walking" OR DE "Eating Behavior" OR DE "Binge Eating" OR DE "Appetite" OR DE "Eating Attitudes" OR DE "Food Intake" OR DE "Mealtimes" OR TI sedentar* OR TI satiety OR TI hunger OR TI hyperphagia OR TI hyperphagic OR TI "energy expenditure" OR TI "energy level" OR AB sedentar* OR AB satiety OR AB hunger OR AB hyperphagia OR AB hyperphagic OR AB "energy expenditure" OR AB "energy level"	Search modes - Boolean/Phrase	133,188
S1	DE "Birth Weight" OR DE "Perinatal Period" OR DE "Prenatal Exposure" OR DE "Prenatal Development" OR TI dohad OR AB dohad OR TI "Perinatal growth" OR TI "infant growth" OR TI "intra-uterine growth" OR TI "intra-uterine programming" OR TI "developmental programming" OR TI "developmental origin" OR TI "developmental origins" OR TI "early-life origin" OR TI "early-life origins" OR TI "catch-up growth" OR TI "accelerated growth" OR TI "Small for Gestational Age" OR TI "dysmaturity" OR AB "Perinatal growth" OR AB "infant growth" OR AB "intra-uterine growth" OR AB "intra-uterine programming" OR AB "developmental programming" OR AB "developmental origin" OR AB "developmental origins" OR AB "early-life origin" OR AB "early-life origins" OR AB "catch-up growth" OR AB "accelerated growth" OR AB "Small for GestaABonal Age" OR AB "dysmaturity"	Search modes - Boolean/Phrase	11,705

#	PsycINFO Query 03-07-2014	Limiters/Expanders	Results
S3	S1 AND S2	Limiters - Publication Type: All Journals, Peer Reviewed Journal, Peer-Reviewed Status-Unknown; Document Type: Journal Article Search modes - Boolean/Phrase	527
S2	DE "Exercise" OR DE "Activity Level" OR DE "Activities of Daily Living" OR DE "Motivation" OR DE "Physical Activity" OR DE "Diets" OR DE "Health Attitudes" OR DE "Lifestyle" OR DE "Active Living" OR DE "Health Behavior" OR DE "Swimming" OR DE "Sports" OR DE "Sports (Attitudes Toward)" OR DE "Motor Performance" OR DE "Jumping" OR DE "Running" OR DE "Walking" OR DE "Eating Behavior" OR DE "Binge Eating" OR DE "Appetite" OR DE "Eating Attitudes" OR DE "Food Intake" OR DE "Mealtimes" OR TI sedentar* OR TI satiety OR TI hunger OR TI hyperphagia OR TI hyperphagic OR TI "energy expenditure" OR TI "energy level" OR AB sedentar* OR AB satiety OR AB hunger OR AB hyperphagia OR AB hyperphagic OR AB "energy expenditure" OR AB "energy level"	Search modes - Boolean/Phrase	150,128
S1	DE "Birth Weight" OR DE "Perinatal Period" OR DE "Prenatal Exposure" OR DE "Prenatal Development" OR TI dohad OR AB dohad OR TI "Perinatal growth" OR TI "infant growth" OR TI "intra-uterine growth" OR TI "intra-uterine programming" OR TI "developmental programming" OR TI "developmental origin" OR TI "developmental origins" OR TI "early-life origin" OR TI "early-life origins" OR TI "catch-up growth" OR TI "accelerated growth" OR TI "Small for Gestational Age" OR TI "dysmaturity" OR AB "Perinatal growth" OR AB "infant growth" OR AB "intra-uterine growth" OR AB "intra-uterine programming" OR AB "developmental programming" OR AB "developmental origin" OR AB "developmental origins" OR AB "early-life origin" OR AB "early-life origins" OR AB "catch-up growth" OR AB "accelerated growth" OR AB "Small for GestaABonal Age" OR AB "dysmaturity"	Search modes - Boolean/Phrase	12,832

#	PsycINFO Query	Limiters/Expanders	Results
S3	S1 AND S2	Limiters - Publication Type: All Journals, Peer Reviewed Journal, Peer-Reviewed Status-Unknown; Document Type: Journal Article Search modes - Boolean/Phrase	548
S2	DE "Exercise" OR DE "Activity Level" OR DE "Activities of Daily Living" OR DE "Motivation" OR DE "Physical Activity" OR DE "Diets" OR DE "Health Attitudes" OR DE "Lifestyle" OR DE "Active Living" OR DE "Health Behavior" OR DE "Swimming" OR DE "Sports" OR DE "Sports (Attitudes Toward)" OR DE "Motor Performance" OR DE "Jumping" OR DE "Running" OR DE "Walking" OR DE "Eating Behavior" OR DE "Binge Eating" OR DE "Appetite" OR DE "Eating Attitudes" OR DE "Food Intake" OR DE "Mealtimes" OR TI sedentar* OR TI satiety OR TI hunger OR TI hyperphagia OR TI hyperphagic OR TI "energy expenditure" OR TI "energy level" OR AB sedentar* OR AB satiety OR AB hunger OR AB hyperphagia OR AB hyperphagic OR AB "energy expenditure" OR AB "energy level"	Search modes - Boolean/Phrase	161,474
S1	DE "Birth Weight" OR DE "Perinatal Period" OR DE "Prenatal Exposure" OR DE "Prenatal Development" OR TI dohad OR AB dohad OR TI "Perinatal growth" OR TI "infant growth" OR TI "intra-uterine growth" OR TI "intra-uterine programming" OR TI "developmental programming" OR TI "developmental origin" OR TI "developmental origins" OR TI "early-life origin" OR TI "early-life origins" OR TI "catch-up growth" OR TI "accelerated growth" OR TI "Small for Gestational Age" OR TI "dysmaturity" OR AB "Perinatal growth" OR AB "infant growth" OR AB "intra-uterine growth" OR AB "intra-uterine programming" OR AB "developmental programming" OR AB "developmental origin" OR AB "developmental origins" OR AB "early-life origin" OR AB "early-life origins" OR AB "catch-up growth" OR AB "accelerated growth" OR AB "Small for GestaABonal Age" OR AB "dysmaturity"	Search modes - Boolean/Phrase	13,497

#	Query	Limiters/Expanders	Results
S3	S1 AND S2	Limiters - Publication Type: All Journals, Peer Reviewed Journal, Peer-Reviewed Status-Unknown; Document Type: Journal Article Search modes - Boolean/Phrase	577
S2	DE "Exercise" OR DE "Activity Level" OR DE "Activities of Daily Living" OR DE "Motivation" OR DE "Physical Activity" OR DE "Diets" OR DE "Health Attitudes" OR DE "Lifestyle" OR DE "Active Living" OR DE "Health Behavior" OR DE "Swimming" OR DE "Sports" OR DE "Sports #Attitudes Toward#" OR DE "Motor Performance" OR DE "Jumping" OR DE "Running" OR DE "Walking" OR DE "Eating Behavior" OR DE "Binge Eating" OR DE "Appetite" OR DE "Eating Attitudes" OR DE "Food Intake" OR DE "Mealtimes" OR TI sedentar* OR TI satiety OR TI hunger OR TI hyperphagia OR TI hyperphagic OR TI "energy expenditure" OR TI "energy level" OR AB sedentar* OR AB satiety OR AB hunger OR AB hyperphagia OR AB hyperphagic OR AB "energy expenditure" OR AB "energy level"	Search modes - Boolean/Phrase	170,849
S1	DE "Birth Weight" OR DE "Perinatal Period" OR DE "Prenatal Exposure" OR DE "Prenatal Development" OR TI dohad OR AB dohad OR TI "Perinatal growth" OR TI "infant growth" OR TI "intra-uterine growth" OR TI "intra-uterine programming" OR TI "developmental programming" OR TI "developmental origin" OR TI "developmental origins" OR TI "early-life origin" OR TI "early-life origins" OR TI "catch-up growth" OR TI "accelerated growth" OR TI "Small for Gestational Age" OR TI "dysmaturity" OR AB "Perinatal growth" OR AB "infant growth" OR AB "intra-uterine growth" OR AB "intra-uterine programming" OR AB "developmental programming" OR AB "developmental origin" OR AB "developmental origins" OR AB "early-life origin" OR AB "early-life origins" OR AB "catch-up growth" OR AB "accelerated growth" OR AB "Small for GestaABonal Age" OR AB "dysmaturity"	Search modes - Boolean/Phrase	14,068

4. Cochrane

#1 Perinatal influences/ DOHaD
Birth weight OR "Prenatal Exposure" OR dohad OR "Perinatal" OR "infant growth" OR "intra-uterine growth" OR "infant programming" OR "fetal programming" OR "intra-uterine programming" OR "developmental programming" OR "intra-uterine plasticity" OR "developmental plasticity" OR "developmental origin" OR "developmental origins" OR "early-life origin" OR "early-life origins" OR "catch-up growth" OR "Small for Gestational Age" OR dysmaturity OR "accelerated growth"

#2 Energy-balance related behavior
Physical acticity - Sedentary behavior - Eating behaviour

"Running" OR "Swimming" OR "Walking" OR "Sports" OR "Life Style" OR "Sedentary Lifestyle" OR "Appetite" OR "Health Behavior" OR "Feeding Behavior" OR "physical activity" OR appetit* OR satiety OR "childhood diet" OR sport* OR exercise* OR "activity level" OR "motor activity" OR "locomotor activity" OR "sedentary" OR sedentarism OR sedentariness OR "physical inactivity" OR "sitting" OR "diet behavior" OR "dietary behavior" OR "feeding behavior" OR "appetitive behavior" OR "diet behaviour" OR "dietary behaviour" OR "feeding behaviour" OR "appetitive behaviour" OR "diet preference" OR "diet preferences" OR "dietary preference" OR "dietary preferences" OR "feeding preference" OR "feeding preferences" OR "feeding practice" OR "feeding practices" OR "diet regulation" OR "feeding regulation" OR "diet control" OR "dietary control" OR "feeding control" OR hunger OR hyperphagia OR hyperphagic OR "energy expenditure" OR "energy level"

Search Name:
Date Run: 03/07/14 11:39:10.627
Description:

ID	Search	Hits
#1	Birth weight or "Prenatal Exposure" or dohad or "Perinatal" or "infant growth" or "intra-uterine growth" or "infant programming" or "fetal programming" or "intra-uterine programming" or "developmental programming" or "intra-uterine plasticity" or "developmental plasticity" or "developmental origin" or "developmental origins" or "early-life origin" or "early-life origins" or "catch-up growth" or "Small for Gestational Age" or dysmaturity or "accelerated growth":ti,ab,kw (Word variations have been searched)	7966
#2	"Running" or "Swimming" or "Walking" or "Sports" or "Life Style" or "Sedentary Lifestyle" or "Appetite" or "Health Behavior" or "Feeding Behavior" or "physical activity" or appetit* or satiety or "childhood diet" or sport* or exercise* or "activity level" or "motor activity" or "locomotor activity" or "sedentary" or sedentarism or sedentariness or "physical inactivity" or "sitting" or "diet behavior" or "dietary behavior" or "feeding behavior" or "appetitive behavior" or "diet behaviour" or "dietary behaviour" or "feeding behaviour" or "appetitive behaviour" or "diet preference" or "diet preferences" or "dietary preference" or "dietary preferences" or "feeding preference" or "feeding preferences" or "feeding practice" or "feeding practices" or "diet regulation" or "feeding regulation" or "diet control" or "dietary control" or "feeding control" or hunger or hyperphagia or hyperphagic or "energy expenditure" or "energy level":ti,ab,kw (Word variations have been searched)	68198
#3	#1 and #2	402

Search Name:
Date Run: 19/05/15 12:18:28.74
Description:

ID Search Hits
#1 Birth weight or "Prenatal Exposure" or dohad or "Perinatal" or "infant growth" or "intra-uterine growth" or "infant programming" or "fetal programming" or "intra-uterine programming" or "developmental programming" or "intra-uterine plasticity" or "developmental plasticity" or "developmental origin" or "developmental origins" or "early-life origin" or "early-life origins" or "catch-up growth" or "Small for Gestational Age" or dysmaturity or "accelerated growth":ti,ab,kw (Word variations have been searched) 8890
#2 "Running" or "Swimming" or "Walking" or "Sports" or "Life Style" or "Sedentary Lifestyle" or "Appetite" or "Health Behavior" or "Feeding Behavior" or "physical activity" or appetit* or satiety or "childhood diet" or sport* or exercise* or "activity level" or "motor activity" or "locomotor activity" or "sedentary" or sedentarism or sedentariness or "physical inactivity" or "sitting" or "diet behavior" or "dietary behavior" or "feeding behavior" or "appetitive behavior" or "diet behaviour" or "dietary behaviour" or "feeding behaviour" or "appetitive behaviour" or "diet preference" or "diet preferences" or "dietary preference" or "dietary preferences" or "feeding preference" or "feeding preferences" or "feeding practice" or "feeding practices" or "diet regulation" or "feeding regulation" or "diet control" or "dietary control" or "feeding control" or hunger or hyperphagia or hyperphagic or "energy expenditure" or "energy level":ti,ab,kw (Word variations have been searched) 76603
#3 #1 and #2 482

Search Name:
Date Run: 05/01/16 14:45:21.756
Description:

ID Search Hits
#1 Birth weight or "Prenatal Exposure" or dohad or "Perinatal" or "infant growth" or "intra-uterine growth" or "infant programming" or "fetal programming" or "intra-uterine programming" or "developmental programming" or "intra-uterine plasticity" or "developmental plasticity" or "developmental origin" or "developmental origins" or "early-life origin" or "early-life origins" or "catch-up growth" or "Small for Gestational Age" or dysmaturity or "accelerated growth":ti,ab,kw (Word variations have been searched) 9571
#2 "Running" or "Swimming" or "Walking" or "Sports" or "Life Style" or "Sedentary Lifestyle" or "Appetite" or "Health Behavior" or "Feeding Behavior" or "physical activity" or appetit* or satiety or "childhood diet" or sport* or exercise* or "activity level" or "motor activity" or "locomotor activity" or "sedentary" or sedentarism or sedentariness or "physical inactivity" or "sitting" or "diet behavior" or "dietary behavior" or "feeding behavior" or "appetitive behavior" or "diet behaviour" or "dietary behaviour" or "feeding behaviour" or "appetitive behaviour" or "diet preference" or "diet preferences" or "dietary preference" or "dietary preferences" or "feeding preference" or "feeding preferences" or "feeding practice" or "feeding practices" or "diet regulation" or "feeding regulation" or "diet control" or "dietary control" or "feeding control" or hunger or hyperphagia or hyperphagic or "energy expenditure" or "energy level":ti,ab,kw (Word variations have been searched) 83424
#3 #1 and #2 545
